# Unveiling high-mobility hot carriers in a two-dimensional conjugated coordination polymer

**DOI:** 10.1038/s41563-025-02246-2

**Published:** 2025-05-13

**Authors:** Shuai Fu, Xing Huang, Guoquan Gao, Petko St. Petkov, Wenpei Gao, Jianjun Zhang, Lei Gao, Heng Zhang, Min Liu, Mike Hambsch, Wenjie Zhang, Jiaxu Zhang, Keming Li, Ute Kaiser, Stuart S. P. Parkin, Stefan C. B. Mannsfeld, Tong Zhu, Hai I. Wang, Zhiyong Wang, Renhao Dong, Xinliang Feng, Mischa Bonn

**Affiliations:** 1 Center for Advancing Electronics Dresden and Faculty of Chemistry and Food Chemistry, Technische Universität Dresden, Dresden, Germany; 2 Max Planck Institute for Polymer Research, Mainz, Germany; 3 Laser Micro/Nano Fabrication Laboratory, School of Mechanical Engineering, Beijing Institute of Technology, Beijing, China; 4 Faculty of Chemistry and Pharmacy, University of Sofia, Sofia, Bulgaria; 5 State Key Laboratory of Metal Matrix Composites, School of Materials Science and Engineering, Future Material Innovation Center, Zhangjiang Institute for Advanced Study, Shanghai Jiao Tong University, Shanghai, China; 6 Center for Advancing Electronics Dresden and Faculty of Electrical and Computer Engineering, TUD Dresden University of Technology, Dresden, Germany; 7 Max Planck Institute of Microstructure Physics, Halle (Saale), Germany; 8 Central Facility for Materials Science Electron Microscopy, Universität Ulm, Ulm, Germany; 9 Nanophotonics, Debye Institute for Nanomaterials Science, Utrecht University, Utrecht, The Netherlands; 10 Department of Chemistry, The University of Hong Kong, Hong Kong, China; 11 Materials Innovation Institute for Life Sciences and Energy (MILES), HKU-SIRI, Shenzhen, China

**Keywords:** Metal-organic frameworks, Coordination polymers, Electronic materials

## Abstract

Hot carriers, inheriting excess kinetic energy from high-energy photons, drive numerous optoelectronic applications reliant on non-equilibrium transport processes. Although extensively studied in inorganic materials, their potential in organic-based systems remains largely unexplored. Here we demonstrate highly mobile hot carriers in crystalline two-dimensional conjugated coordination polymer Cu_3_BHT (BHT, benzenehexathiol) films. Leveraging a suite of ultrafast spectroscopic and imaging techniques, we map the microscopic charge transport landscape in Cu_3_BHT films following non-equilibrium photoexcitation across temporal, spatial and frequency domains, revealing two distinct high-mobility transport regimes. In the non-equilibrium regime, hot carriers achieve an ultrahigh mobility of ~2,000 cm^2^ V^–1^ s^–1^, traversing grain boundaries up to ~300 nm within a picosecond. In the quasi-equilibrium regime, free carriers exhibit Drude-type, band-like transport with a remarkable mobility of ~400 cm^2^ V^–1^ s^–1^ and an intrinsic diffusion length exceeding 1 μm. These findings position two-dimensional conjugated coordination polymers as versatile platforms for advancing organic-based hot carrier applications.

## Abstract (web summary)

Hot carrier transport in organic systems has remained elusive due to rapid energy relaxation and limited transport properties. Here highly mobile hot carriers and their relaxation dynamics are reported in a crystalline two-dimensional conjugated coordination polymer, revealing two distinct transport regimes.

## Main

Hot carriers refer to high-energy electrons and holes out of thermal equilibrium with the crystal lattice. They exhibit fascinating properties that have catalysed innovations in various fields, including photovoltaics, transistors, photodetectors, photocatalysis and bolometers^1–5^. Recent computational and experimental studies^6–14^ have delineated a generalized three-stage fate for hot carriers: generation, thermalization and relaxation. In stark contrast to the growing understanding of hot carriers in inorganic or hybrid materials, the study of hot carriers in organic compounds lags far behind. This gap primarily arises from the ultrafast energy relaxation process in conventional organic compounds^15,16^, compounded by their limited charge transport capabilities due to pervasive dynamic disorder, strong Coulomb interactions between electron–hole pairs and intense charge–vibration coupling^7,17–19^. As a result, organic compounds face substantial challenges in achieving viability for organic-based hot carrier applications, which are actively sought after in the field.

**Fig. 1:**
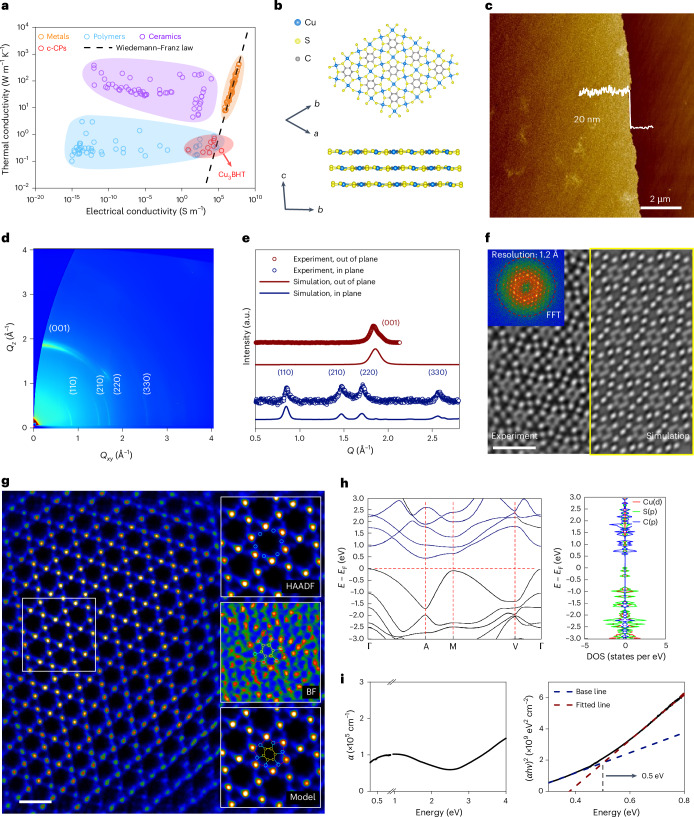
Characterization of Cu_3_BHT films synthesized by liquid–liquid interfacial reaction. **a**, Ashby plot illustrating thermal conductivity versus electrical conductivity for various material systems. **b**, Crystal structure of Cu_3_BHT viewed along the stacking direction (top) and from the side (bottom). **c**, AFM image of the Cu_3_BHT film on a fused silica substrate. **d**, 2D GIWAXS image of Cu_3_BHT. **e**, Experimental and calculated GIWAXS intensity profiles of Cu_3_BHT, projected along the in-plane and out-of-plane directions. **f**, AC-HRTEM image and the corresponding fast Fourier transform (FFT) pattern of Cu_3_BHT (left) and its simulated image (right), denoised using Wiener filtering. Scale bar, 1 nm. **g**, Atomic-resolution high-angle annular dark-field scanning transmission electron microscopy image of Cu_3_BHT. Scale bar, 1 nm. The three panels on the right display the high-angle annular dark-field (HAADF), bright-field (BF) and model images, respectively. **h**, Calculated electronic band structure (left) and projected density of states (DOS; right) of Cu_3_BHT with a Cu^2+^/Cu^+^ ratio of 0:3. **i**, Optical absorption spectrum of Cu_3_BHT film (left) and data in the infrared range are plotted using Tauc units (right). The vertical black dashed line at 0.5 eV indicates the energy corresponding to the intersection of the base line and the fitted line.

The recent rise in synthetic organic 2D crystals, particularly 2D conjugated coordination polymers (2D c-CPs) or 2D conjugated metal–organic frameworks, may bridge this gap^20^. These 2D c-CPs exhibit appealing (opto-)electronic properties due to tunable intralayer *d*–*π* conjugation and interlayer electronic coupling^20–22^. Recent advances in synthesis strategies have allowed precise control over stoichiometry, structural topology, layer orientation and morphology, giving rise to a host of exquisite structures with diverse properties^23,24^. Two striking properties that stand out are their high electrical conductivity, up to 10^3^ S cm^−1^ (refs. ^25,26^), on par with metals, and their low thermal conductivity below 1 W m^−1^ k^−1^ (ref. ^27^), akin to linear polymers (Fig. 1a and Supplementary Tables 1−4). These two seemingly contradictory characteristics—functioning as electrical conductors and being thermal insulators—bring 2D c-CPs to the forefront of thermoelectric and hot carrier applications. The thermoelectric behaviour is reminiscent of the phonon glass–electron crystal paradigm, which is crucial for achieving a high thermoelectric figure of merit^28,29^. In the context of hot carrier applications, 2D c-CPs offer the opportunity to engineer electronic and phononic properties at the molecular scale, enabling high-mobility charge transport channels and simultaneously decelerating hot carrier cooling processes^21,30–32^. Despite these promising prospects, no study, to the best of our knowledge, has yet observed the signatures of hot carriers in 2D c-CPs, let alone evaluated their transport characteristics and discovered high-mobility non-equilibrium electronic states that are paramount for hot carrier applications.

Here we combine time-resolved terahertz spectroscopy (TRTS), transient absorption spectroscopy (TAS) and transient absorption microscopy (TAM) to visualize the spatiotemporal evolution of non-equilibrium photoexcitation and track the excess-kinetic-energy-dependent charge transport properties in a model 2D c-CP system, namely, Cu_3_BHT (BHT, benzenehexathiol; Fig. 1b). Jointly, these experiments provide a comprehensive understanding of transport phenomena across temporal, spatial and frequency domains. Our findings reveal that above-bandgap photoexcitation launches a transport cascade in highly crystalline Cu_3_BHT films. Hot carriers dominate the non-equilibrium regime, exhibiting exceptionally high charge mobility of ~2,000 cm^2^ V^–1^ s^–1^ and migrating up to ~300 nm across grain boundaries. Owing to the low optical phonon energy and small electron–hole reduced effective mass, the cooling of these highly mobile hot carriers occurs on relatively long timescales, up to ~750 fs, comparable with state-of-the-art lead-halide perovskites known for their suitability for hot carrier applications^33^. After cooling and entering the quasi-equilibrium regime, band-edge carriers exhibit band-like Drude-type free carrier transport with an impressive charge mobility of ~400 cm^2^ V^–1^ s^–1^ and a remarkably long intrinsic diffusion length exceeding 1 μm.

## Synthesis and characterization of Cu_3_BHT films

Cu_3_BHT films were synthesized via an optimized liquid–liquid interfacial synthesis method^25,26^ (Methods). Atomic force microscopy and Raman characterization confirm the coordination between Cu^2+^ and BHT ligands at the water–toluene interface, forming a large-area Cu_3_BHT film with a thickness of ~20 nm and a root mean square roughness of 1.3 nm (Fig. 1c and Supplementary Figs. 1 and 2). Aberration-corrected high-resolution transmission electron microscopy (AC-HRTEM; Fig. 1f, left) reveals a highly ordered lattice with atomic resolution (~1.2 Å) and a lattice spacing of ~0.73 nm. High-angle annular dark-field scanning transmission electron microscopy (Fig. 1g) visualizes a high-symmetry, non-distorted kagome lattice formed by Cu atoms: each BHT unit connects to six neighbouring BHT units via shared Cu atoms; each Cu atom coordinates with four S atoms, forming a dense hexagonal *d*–*π* conjugated plane. Grazing-incidence wide-angle X-ray scattering (GIWAXS; Fig. 1d,e) indicates a preferential face-on orientation with an interlayer distance of 3.4 Å. On the basis of AC-HRTEM and GIWAXS analyses, we proposed a triclinic lattice structure, featuring a slipped-AA-stacking geometry and unit-cell parameters of *a* = *b* = 8.675 Å, *c* = 3.489 Å, *α* = *β* = 99.94° and *γ* = 60.12°. The simulated AC-HRTEM image (Fig. 1f, right) and GIWAXS diffraction signals (Supplementary Fig. 3), based on this model, align well with the experimental observations.

X-ray photoelectron spectroscopy, X-ray absorption near-edge structure and extended X-ray absorption fine structure results indicate a fractional Cu oxidation state and a square planar coordination geometry (Supplementary Figs. 4−6), which can be attributed to an intramolecular pseudo-redox mechanism between Cu^+^/Cu^2+^ and BHT ligands^34,35^. Density functional theory calculations (see the computational details in the Supplementary Information) reveal that gradually reducing the Cu^2+^/Cu^+^ ratio from 1 to 0 induces a surprising trend of bandgap opening (Fig. 1h and Supplementary Fig. 7). For instance, Cu_3_BHT with a Cu^2+^/Cu^+^ ratio of 0:3 shows strongly dispersive energy bands and a bandgap of ~0.4 eV. Optical absorption measurements reveal a broadband absorption feature, with the corresponding Tauc plot indicating an absorption edge of ~0.5 eV (Fig. 1i). This gapped nature is further validated by ultraviolet photoelectron spectroscopy and variable-temperature conductivity measurements (Supplementary Figs. 8 and 9). Note that although the room-temperature electrical conductivity (~48 S cm^−1^) of the synthesized Cu_3_BHT film surpasses most 2D c-CPs^23^, it is substantially lower than that of metallic-phase Cu_3_BHT bulk crystals (up to 2,500 S cm^−1^)^26^. Unlike semiconducting thin films, these bulk metallic crystals feature an AB-stacking mode and interlayer Cu–S covalent bonds^36^. The distinct electrical behaviours of Cu_3_BHT reported in different studies may be attributed to the thickness-dependent properties, resulting from the unique growth mechanism of liquid–liquid interfacial synthesis: during the early stages of the reaction, interfacial confinement keeps BHT ligands lying flat at the liquid–liquid interface, promoting the formation of highly ordered face-on oriented layers without interlayer Cu–S bonds. As the film thickens, this interfacial confinement effect weakens, leading to disordered stacking in later-formed layers and the formation of interlayer Cu–S bonds. Such thickness-dependent structural variations, together with the potential crystallinity evolution, may explain the transition from semiconducting behaviour in Cu_3_BHT thin films and monolayers^37,38^ to metallic behaviour in Cu_3_BHT bulk crystals and thick films^26,36^.

## Highly mobile hot carriers and hot phonon bottleneck

**Fig. 2:**
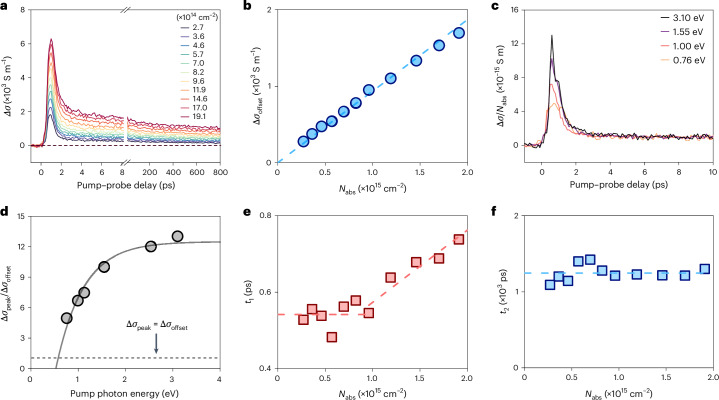
Non-equilibrium photoexcitation cascades and hot phonon bottleneck in Cu_3_BHT. **a**, Terahertz photoconductivity dynamics following 1.55 eV excitation at different absorbed photon fluences (*N*_abs_). The *N*_abs_ range used is 2.7–19.1 × 10^14 ^cm^–2^, which corresponds to a three-dimensional density range of 1.4–9.6 × 10^20 ^cm^–3^. **b**, Δ*σ*_offset_, defined as the average THz photoconductivity between 6 ps and 8 ps, at different *N*_abs_ values. The dashed line represents a linear fit through the origin of the coordinate system. **c**, Pump photon energy (*hv*)-dependent THz photoconductivity dynamics normalized by *N*_abs_. **d**, Ratio of the maximum photoconductivity (Δ*σ*_peak_) to the average THz photoconductivity between 6 ps and 8 ps (Δ*σ*_offset_) as a function of photon energy *hv*. The solid grey line represents the extrapolation used to estimate the critical photon energy value at which the ratio equals 1 (dashed line). **e**,**f**, Time constants associated with hot carrier cooling *t*_1_ (**e**) and charge recombination *t*_2_ (**f**) versus *N*_abs_ inferred from the data in **a**. The dashed lines are guides to the eye.

We use TRTS as a contact-free approach to explore the microscopic charge transport properties in Cu_3_BHT films. In TRTS measurements, an ultrashort pump pulse (~50 fs duration) with tunable photon energy photoinjects hot carriers into the sample via above-gap excitations. Subsequently, a time-delayed, single-cycle terahertz (THz) electromagnetic pulse (~1 ps duration) propagates through the sample, driving the photogenerated carriers over short distances (typically sub-tens to tens of nanometres), affording insights into their intracrystal charge transport properties^39^ (Methods). Figure 2a shows the pump-fluence-dependent THz photoconductivity dynamics following 1.55 eV excitation. The non-resonant excitation induces a sub-picosecond rise in photoconductivity due to the quasi-instantaneous generation of mobile carriers. The positive THz photoconductivity further corroborates the intrinsic semiconducting nature of the synthesized Cu_3_BHT films^30^. This is followed by a photoconductivity decay characterized by a fast-decay component within ~1 ps and a long-lived component persisting for over ~1 ns. Since the photoconductivity Δ*σ*(*t*) is determined by the product of the photogenerated carrier density (*n*), elementary charge (*e*) and electron–hole sum mobility (*μ*) as Δ*σ*(*t*) *=* *neμ*, two possible scenarios can account for the fast-decay component: (1) hot carriers rapidly lose their excess kinetic energy and populate band-edge states within the instrument’s time resolution (less than ~50 fs), with the fast-decay component reflecting a reduction in band-edge carrier density due to fast charge localization or trapping (*n* decays quickly after photoexcitation)^40,41^; (2) hot carriers possess much higher charge mobility than band-edge carriers and energy relaxation occurs on a long timescale relative to the instrument’s time resolution (greater than ~50 fs), with the fast-decay component signifying a decrease in charge mobility as the non-equilibrium electronic system relaxes (*μ* drops quickly following photoexcitation and hot carrier relaxation)^42^. For clarity, we define the maximum photoconductivity as Δ*σ*_peak_ and the average photoconductivity value between 6 ps and 8 ps as Δ*σ*_offset_. A key criterion for distinguishing these two scenarios is the dependence of Δ*σ*_offset_ on the absorbed photon density (*N*_abs_). As shown in Fig. 2b, the linear dependence of Δ*σ*_offset_ on *N*_abs_ (1) excludes the dominant role of the defect trapping scenario, which would otherwise exhibit a characteristic super-linear dependence^40,41^; (2) demonstrates that band-edge carrier density increases linearly with *N*_abs_, without hitting the threshold for absorption saturation or non-radiative Auger relaxation; and (3) indicates that *μ* of the band-edge carriers remains constant, with negligible carrier–carrier interactions within the investigated *N*_abs_ range.

To further validate that cooling of high-mobility hot carriers drives the fast decay, we varied the excitation photon energy (*hv*), and performed TRTS measurements in the low-*N*_abs_ limit (<1 × 10^14 ^cm^−2^) to minimize the scattering between hot carriers and hot phonons (Supplementary Fig. 10). Figure 2c compares the photoconductivity response normalized to *N*_abs_ (Δ*σ*/*N*_abs_), following optical excitation at different *hv* values. Within the first 10 ps time in which *n* can be considered constant, Δ*σ*/*N*_abs_ provides a direct view of the temporal evolution of *μ*. Qualitatively, we find that a higher *hv* results in an increased transient *μ* within the first picosecond, after which *μ* drops substantially until reaching the same value regardless of *hv*. The extrapolation of Δ*σ*_peak_/Δ*σ*_offset_ to *hv* (Fig. 2d and Methods) suggests that the critical photon energy corresponding to resonant excitation is ~560 ± 50 meV, consistent with the absorption edge revealed by Tauc analysis. These results indicate that hot carriers exhibit a much higher *μ* than band-edge carriers in Cu_3_BHT. Accordingly, the subsequent long-lived component can be assigned to the recombination process involving band-edge electrons and holes in the quasi-equilibrium regime.

By extracting the time constants associated with hot carrier cooling (*t*_1_) and band-edge carrier recombination (*t*_2_) at different *N*_abs_ values using a biexponential decay function, we find that *t*_1_ exhibits a stepwise variation with *N*_abs_, whereas *t*_2_ remains unchanged, irrespective of *N*_abs_ (Fig. 2e and Supplementary Fig. 11). Specifically, *t*_1_ stays at ~500 fs when *N*_abs_ is below 10^15 ^cm^−2^. However, once *N*_abs_ surpasses this threshold, *t*_1_ gradually increases to ~750 fs as *N*_abs_ rises, a hallmark of the hot phonon bottleneck^43^. On the other hand, the invariance of *t*_2_ ≈ 1.2 ns across the studied *N*_abs_ range indicates that trap-assisted recombination is probably the dominant recombination mechanism. The observed hot phonon bottleneck can be understood by noting the phonon dispersion in Cu_3_BHT (ref. ^44^), where the low-energy optical phonon branches at around 10 meV intersect with the acoustic phonon branches. The low optical phonon energy and small electron–hole reduced effective mass favour reduced energy dissipation rates^12^. This results in a relatively long hot carrier lifetime in Cu_3_BHT, superior to conventional organic compounds (typically below 100 fs)^15,16^ and comparable with inorganic and hybrid perovskite materials (ranging from 200 fs to a few picoseconds)^33^. Furthermore, the considerable phononic overlap, combined with the limited phonon propagation due to the low thermal conductivity of Cu_3_BHT, may facilitate the upconversion of acoustic phonons to optical phonons, which is responsible for the observed hot phonon bottleneck^45^.

## Crossover from non-equilibrium to quasi-equilibrium regime

**Fig. 3:**
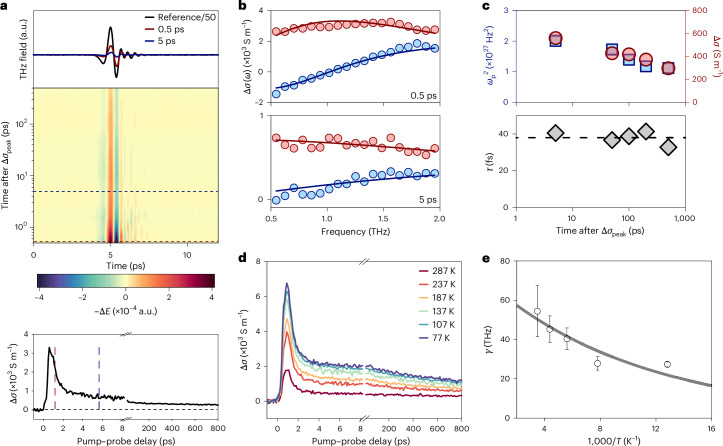
Spectral signature of the transition from hot carriers to band-edge carriers. **a**, Top, time-resolved THz electric field transmitted through unexcited Cu_3_BHT and the pump-induced time-resolved THz electric field changes at representative times after Δ*σ*_peak_. Middle: pseudo-colour plot of the pump-induced time-resolved THz electric field changes at different times after Δ*σ*_peak_. The results are collected under 1.55 eV photoexcitation at *N*_abs_ = 0.6 × 10^15^ cm^–2^ at room temperature. Bottom, dynamics of the real part of THz photoconductivity. **b**, Frequency-resolved complex THz photoconductivity measured at 0.5 ps (top) and 5 ps (bottom) after Δ*σ*_peak_. The red and blue solid lines are the Drude–Smith (top) and Drude (bottom) fits, describing the real and imaginary components of the complex THz photoconductivity. **c**, Top: squared plasma frequencies (blue squares, left *y* axis) inferred from the Drude fits and the photoconductivity (red circles, right *y* axis) at different times after Δ*σ*_peak_. Bottom: charge scattering times inferred from the Drude fits at different times after Δ*σ*_peak_. **d**, Temperature-dependent THz photoconductivity dynamics. The results are collected under 1.55 eV photoexcitation at *N*_abs_ = 0.4 × 10^15^ cm^−2^. **e**, Charge scattering rates at different temperatures. The circles and error bars are the mean values and standard error, respectively, derived from the Drude fits to the temperature-dependent Δ*σ*(*ω*) data (Supplementary Fig. 12). The solid line is a fit according to the Arrhenius relation.

We further track time-varying THz waveforms at various time delays after reaching Δ*σ*_peak_ (Fig. 3a), from which we can infer the temporal evolution of the frequency-resolved complex THz photoconductivity Δ*σ*(*ω*); the dynamics of the real part of the THz photoconductivity is shown in the bottom panel. We observe that Δ*σ*(*ω*) exhibits distinct frequency dispersions in the non-equilibrium and quasi-equilibrium regimes (Fig. 3b). Specifically, in the non-equilibrium regime, Δ*σ*(*ω*) shows suppressed real photoconductivity and negative imaginary photoconductivity at low frequencies. These spectral features reflect charge transport described by the phenomenological Drude–Smith model (Methods), where charge localization induced by backscattering events hinders long-range charge migration. The backscattering probability is quantified by the parameter *c*, which ranges from 0 (isotropic scattering) to −1 (complete backscattering)^46^. By contrast, in the quasi-equilibrium regime, Δ*σ*(*ω*) shows positive real and imaginary components that converge with increasing frequency, indicative of delocalized free carrier transport, as described by the Drude model:

$$\Delta \sigma \left(\omega \right)=\frac{n{e}^{2}\tau }{{m}^{* }\left(1-{\rm{i}}\omega \tau \right)},\,n=\frac{{\omega }_{{\rm{p}}}^{2}{{m}^{* }\varepsilon }_{0}}{{e}^{2}},$$

where *τ*, m*, *ω*_p_ and *ε*_0_ represent the momentum-averaged charge scattering time, electron–hole reduced effective mass, plasma frequency and vacuum permittivity, respectively. The evolution of frequency dispersion during the transition from hot carriers to band-edge carriers can be understood as follows: hot carriers, with excess kinetic energy, can more easily navigate the fluctuating energy landscape and travel relatively long distances exceeding the grain size (~100 nm), thereby encountering a higher probability of backscattering (for example, at grain boundaries) than band-edge carriers. Fitting Δ*σ*(*ω*) in the quasi-equilibrium regime with the Drude model yields *τ* of ~41 ± 3 fs. Using m* = 0.187*m*_0_ from the density functional theory calculations, *µ* of band-edge carriers in Cu_3_BHT is estimated to be 405 ± 30 cm^2^ V^–1^ s^–1^ in the d.c. limit, following *µ* = *eτ*/m*. By knowing the carrier lifetime *t*_2_ and mobility, we estimate the intrinsic diffusion length of the band-edge carriers to be ~1,100 ± 300 nm (Methods). By further considering the photoconductivity ratio between the non-equilibrium and quasi-equilibrium states under the photoexcitation conduction used, the *µ* value of hot carriers generated with *hv* = 1.55 eV at Δ*σ*_peak_ is inferred to be approximately 2,000 cm^2^ V^–1^ s^–1^. These values set new records for both mobility and diffusion length in organic materials (Supplementary Table 5). The Drude fits to Δ*σ*(*ω*) at different time delays in the quasi-equilibrium regime reveal the temporal evolution of microscopic parameters (for example, *n* and *τ*) related to charge transport during recombination. As shown in Fig. 3c, ${\omega }_{{\rm{p}}}^{2}$ (proportional to *n*) follows the same trend as Δ*σ* over time, indicating that the decrease in *n* drives the photoconductivity decay, as a result of (probably trap-assisted) recombination. Meanwhile, *τ* remains largely unchanged in the quasi-equilibrium regime, consistent with a delocalized charge transport picture of a dilute free electron gas free from strong carrier–carrier interactions^47^.

Temperature-dependent measurements reveal substantially increased Δ*σ*_peak_ and Δ*σ*_offset_ as temperature decreases (Fig. 3d), where Δ*σ*(*ω*) measured in the quasi-equilibrium regime retains the spectral features of Drude-type transport (Supplementary Fig. 12). The inferred scattering rate *γ* = 1/*τ* increases from ~30 THz at 78 K to ~50 THz at 287 K (Fig. 3e), indicating that freezing out phonons or vibrational modes effectively reduces charge scattering. The positive temperature coefficient of *γ* is a hallmark of band-like transport. The temperature–*γ* relationship reveals a critical energy value of 8 ± 2 meV (65 ± 15 cm^–1^) following the Arrhenius relation (Methods), pointing to a phonon mode of approximately that energy involved in charge scattering. This value closely matches the observed Raman peak centred at ~8 meV (Supplementary Fig. 2), attributed to Cu and S atomic vibrations with pronounced electron–phonon coupling^36^.

## Spatiotemporal and energetic evolution

**Fig. 4:**
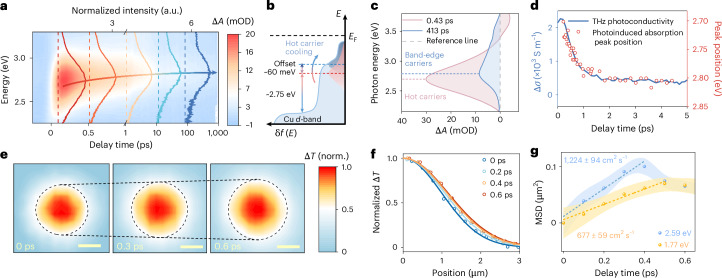
Temporal and energetic evolution of hot carrier cooling and visualization of hot carrier transport in real space. **a**, Pseudo-colour 2D image of TA as a function of probe photon energy and delay time under 1.77 eV photoexcitation at *N*_abs_ = 1.1 × 10^15^ cm^–2^. The coloured solid lines represent the corresponding normalized TA spectra at representative pump–probe delay times (that is, 0.15 ps, 0.5 ps, 1 ps, 10 ps and 100 ps). **b**, Schematic of hot carrier cooling as the origin of the blueshift. **c**, Global fitting analysis deconvolutes the respective spectral contributions from hot and band-edge carriers. **d**, Comparison of THz photoconductivity decay and TA signal peak energy. **e**, Representative pseudo-colour 2D images at different pump–probe delay times under 1.77 eV photoexcitation at *N*_abs_ = 0.7 × 10^15^ cm^–2^. Scale bar, 1 μm. **f**, Spatial profiles (dots) and Gaussian fits (curves) of photogenerated charge carriers at different pump–probe delay times. **g**, Determination of hot carrier diffusion coefficients for two *hv* values at *N*_abs_ = 0.7 × 10^15^ cm^–2^. Each solid circle represents the variance in carrier distribution, and the error bars denoted by the shaded areas are the standard error obtained from fitting the variance. The dashed lines are linear fits used to extract the diffusion constants under different photoexcitation conditions.

Next, we explore photogenerated carriers’ spatiotemporal and energetic evolution across different transport regimes using TAS and TAM (Methods). Figure 4a presents a pseudo-colour 2D image of Cu_3_BHT, along with the normalized cross-section transient absorption (TA) spectra at representative pump–probe delay times under 1.77 eV photoexcitation. The spectra exhibit a broad photoinduced absorption signal spanning the 2.4–3.0 eV range, with a noticeable blueshift of ~60 meV within the first few picoseconds (Fig. 4a and Supplementary Fig. 13). Similar spectral signatures are also observed at higher *hv* excitations (Supplementary Fig. 14). We find that the temporal evolution of the photoinduced absorption band mirrors Δ*σ* dynamics, suggesting that the observed spectral blueshift is associated with hot carrier cooling^13,48^. This interpretation is further supported by the calculated electronic band structure and density of states, where transitions from the Cu *d*-band to the valence band edge can rationalize the observed photoinduced absorption signal (Fig. 4b and Supplementary Fig. 7). To consolidate this, we perform a global fitting analysis of the TA spectra, identifying two distinct spectral components centred at 2.69 eV and 2.79 eV, respectively (Fig. 4c). In particular, the decay of the low-energy component coincides with the rise in the high-energy component on the picosecond timescale, whereas the high-energy component persists into the nanosecond timescale (Supplementary Fig. 15). Given that their dynamic signatures correspond closely to those of hot carriers and band-edge carriers elucidated by TRTS, we assign the low-energy and high-energy components to hot carriers and band-edge carriers, respectively. Figure 4d shows that the peak energy shift signifying hot carrier relaxation evolves remarkably in tandem with the photoconductivity decay, substantiating the blueshift as a spectroscopic indicator of hot carrier cooling. The evolution of the carrier temperature yields a consistent trend between the extracted carrier temperature and Δ*σ* (Supplementary Fig. 16), providing additional evidence for the high-mobility nature of hot carriers.

The time-evolving spatial distribution of the photoexcited population, as revealed by TAM, is quantitatively characterized by the mean square displacement (MSD = ${s}_{t}^{2}$ – ${s}_{0}^{2}$), where *s* is the Gaussian width of the population profile at a delay time *t*. Representative pseudo-colour 2D images of the signal intensity across spatial locations are shown in Fig. 4e. During the first ~0.6 ps, photogenerated carriers undergo ultrafast expansion, as depicted in the 2D image cross-sections (Fig. 4f) and the extracted spatiotemporal profiles (Fig. 4g). By increasing *hv* from 1.77 eV to 2.59 eV to raise the hot carrier temperature and maintaining constant *N*_abs_ below the 10^15 ^cm^–2^ threshold (Fig. 2e), we observe an increase in the diffusion coefficient (*D*) from 677 ± 59 cm^2 ^s^–1^ to 1,224 ± 94 cm^2 ^s^–1^. The ultrafast diffusion timescale, exceptional charge transport properties and enhanced charge transport properties at higher *hv* are consistent with the hot carrier characteristics identified by TRTS (Fig. 2c). Note that the *D* value of hot carriers observed in Cu_3_BHT films is superior to that of the dominant charge species reported in other material systems^9,49,50^ (Supplementary Table 6). Together, these findings demonstrate—from two complementary perspectives—that the excess kinetic energy of hot carriers substantially boosts the charge transport properties. Furthermore, under the same 1.77 eV excitation, the *D* value of hot carriers decreases monotonously as *N*_abs_ increases (Supplementary Fig. 17), which parallels the reduction in *µ* of hot carriers with rising *N*_abs_ (Supplementary Fig. 10), probably due to the more pronounced scattering at elevated hot phonon populations. Depending on the photoexcitation conditions, the hot carrier propagation length (*l*_*t*_) can be estimated to range from 200 nm to 320 nm, following ${l}_{t}=\,\sqrt{{s}_{t}^{2}-{s}_{0}^{2}}$ (ref. ^51^). These long propagation lengths, far exceeding the average grain size, indicate the cross-boundary transport behaviour of hot carriers. The ultrafast diffusion regime is followed by a contracting phase, which can tentatively be attributed to a consequence of a hot phonon bottleneck, as further justified by *N*_abs_-dependent and *hv*-dependent measurements (Supplementary Fig. 18). This also rationalizes the earlier onset of the contraction phase at higher *hv* or *N*_abs_ (Fig. 4g and Supplementary Fig. 10).

On a timescale of tens of picoseconds, we identify a slower diffusion phase that can be attributed to the quasi-equilibrium transport of band-edge carriers (Supplementary Fig. 17). Following 1.77 eV photoexcitation, the *D* value of band-edge carriers ranges from 1.1 ± 0.2 cm^2 ^s^–1^ to 1.9 ± 0.3 cm^2 ^s^–1^ (corresponding to ambipolar mobility (*µ*_a_) from 42 ± 8 cm^2^ V^–1^ s^–1^ to 72 ± 12 cm^2^ V^–1^ s^–1^), with an increasing trend as *N*_abs_ increases. The relatively low mobility and its distinct dependence on *N*_abs_, compared with those inferred from TRTS, can be reconciled by the different transport length scales each technique probes: TRTS characterizes local, intrinsic and intracrystal transport with minimal influence from grain boundaries, featuring constant mobility when carrier–carrier interactions are negligible; TAM encompasses both intracrystal and intercrystal transport, exhibiting lower mobility that increases with elevated equilibrated electron–lattice temperatures due to the more favourable thermally activated hopping transport across grain boundaries^52^.

## Outlook

In this work, we use complementary ultrafast techniques to elucidate how energy relaxation couples with charge transport in 2D c-CPs. The first demonstration of high-mobility non-equilibrium states in 2D c-CPs opens up new possibilities for advancing novel organic-based (opto-)electronic applications, including (1) hot electron transistors, where hot carriers can traverse crystal lattices ballistically with near-zero base transit time; (2) hot carrier photovoltaics, where the excess kinetic energy of hot carriers can be harnessed to enhance open-circuit voltages and short-circuit currents; and (3) plasmonic photocatalysis, where the non-equilibrium nature of hot carriers can drive catalytic reactions that are otherwise unattainable under equilibrium conditions. Furthermore, the impressive *µ* value in the quasi-equilibrium regime motivates further exploration, such as Hall and quantum Hall measurements. The potential of this material is further reinforced by the extensive chemical and structural tunability of 2D c-CPs, along with recent breakthroughs in the fabrication of large crystals and ultrasmooth films for device integration^22,53^. Overall, these findings only scratch the surface, with many opportunities to be explored through metal substitution and diversification, ligand design and guest molecule interactions.

## Methods

### Synthesis of BHT

BHT was synthesized via a modified literature method^54^. As illustrated in Supplementary Information, scheme 1, 1,2,3,4,5,6-hexakis(benzylthio)benzene (BHT-6Bn) was first prepared by reacting hexachlorobenzene with benzyl mercaptan in dimethylformamide at room temperature for 8 h. Traditional deprotection via Birch reduction presents drawbacks, including the use of hazardous sodium–ammonia solutions and the formation of a highly oxidation-sensitive, fully deprotonated intermediate. To circumvent these issues, we used boron tribromide (BBr_3_) in fluorobenzene at 60 °C for 48 h, which facilitated the moderate cleavage of benzyl-protecting groups and forming a stable BHT(BBr_3_)_3_ intermediate. Subsequent hydrolysis with methanol at room temperature for 30 min yielded BHT as a white powder.

### Synthesis of Cu_3_BHT films

Referring to previous reports^25,26^, Cu_3_BHT films are synthesized via an interfacial reaction between two immiscible liquid media, namely, CuSO_4_/H_2_O and BHT/toluene. CuSO_4_ and toluene were purchased from Sigma-Aldrich. Water was purified using a Milli-Q system. Both solvents were degassed via the freeze–thaw method before use. First, the substrate of interest is placed into an empty beaker before synthesis. Then, 30 ml of CuSO_4_ aqueous solution (0.5 mg ml^–1^) and 30 ml of toluene are injected into the beaker in sequence, serving as the Cu^2+^ source and buffer layer, respectively. After forming a stable liquid–liquid interface, 2 ml of BHT in a toluene solution (0.1 mg ml^–1^) is gently injected into the buffer layer to initiate the coordination polymerization between Cu^2+^ and BHT at the interface. The formation of a dark-coloured film at the liquid–liquid interface can be observed with the naked eye. After film formation, the liquid is gently removed using a syringe, allowing the formed film to settle naturally onto the target substrate. The obtained film is washed sequentially with methanol and acetone to remove potential impurities. Finally, the film is dried overnight under ambient conditions.

### TRTS

TRTS was used to track the time- and frequency-resolved photoconductivities of Cu_3_BHT films. The setup was powered by a Ti:sapphire mode-locked regenerative amplifier, which delivered ultrashort ~50 fs laser pulses centred at 1.55 eV, with a repetition rate of 1 kHz. Optical excitations at 1.55 eV and 3.10 eV were achieved by directly using a branch of the fundamental 1.55 eV laser pulse and by frequency doubling it with the aid of a β-BiB_3_O_6_ crystal. Optical excitations at other photon energies were obtained using a commercial optical parametric amplifier (LIGHT CONVERSION). Single-cycle THz radiation with a duration of ~1 ps was generated and detected using a pair of 1-mm-thick (110)-oriented ZnTe crystals through optical rectification and free-space electro-optic sampling, respectively. Measurements at room temperature were performed in the transmission mode in a dry N_2_-purged environment, whereas temperature-dependent measurements were carried out by placing the Cu_3_BHT film in a cryostat under vacuum conditions (pressure below 1 × 10^−4^ mbar). The time-resolved photoconductivity was measured by fixing the sampling beam to the peak of the transient THz electric field and recording the pump-induced signal intensity changes when varying the relative time delay between the pump pulse and the THz probe. The frequency-resolved complex THz photoconductivity Δ*σ*(*ω*) was accessed by recording the time-varying THz profiles transmitted through the Cu_3_BHT film with and without optical excitation (*E*′(*t*) and *E*(*t*)), applying Fourier transform (*E*′(*ω*) and *E*(*ω*)), and adopting the thin-film approximation:

$$\Delta \sigma \left(\omega \right)=-\frac{{n}_{1}+{n}_{2}}{{Z}_{0}l}\left(\frac{{E}^{{\prime} }\left(\omega \right)-E\left(\omega \right)}{E\left(\omega \right)}\right),$$

where *Z*_0_ = 377 Ω is the impedance of free space; *n*_1_ and *n*_2_ are the refractive indices of the media before and after the Cu_3_BHT film, respectively; and *l* is the Cu_3_BHT film thickness. To estimate the critical photon energy corresponding to resonant excitation (*E*_g_), we fit $\frac{\Delta {\sigma }_{{{\rm{peak}}}}}{\Delta {\sigma }_{{{\rm{offset}}}}}$ to *hv* using the following equation:

$$\frac{\Delta {\sigma }_{{\rm{peak}}}}{\Delta {\sigma }_{{\rm{offset}}}}=A\left(1-{{\rm{e}}}^{-\frac{hv-{E}_{{\rm{g}}}}{{E}_{{\rm{g}}}}}\right)+1,$$

where *A* is the pre-factor. When *hv* is equal to *E*_g_, $\frac{\Delta {\sigma }_{{{\rm{peak}}}}}{\Delta {\sigma }_{{{\rm{offset}}}}}=1$, reflecting the long-lived nature of band-edge carriers. When *hv* is much larger than 1, $\frac{\Delta {\sigma }_{{{\rm{peak}}}}}{\Delta {\sigma }_{{{\rm{offset}}}}}$ tends to converge to a finite value, consistent with the fact that the electronic temperature gradually approaches saturation with increasing input energy.

The Drude–Smith model describing spatially confined charge transport of hot carriers reads:

$$\Delta \sigma \left(\omega \right)=\frac{{\omega }_{{\rm{p}}}^{2}{\varepsilon }_{0}{\tau }_{{{\rm{DS}}}}}{1-{\rm{i}}\omega {\tau }_{{{\rm{DS}}}}}\left(1+\frac{c}{1-{\rm{i}}\omega {\tau }_{{{\rm{DS}}}}}\right),$$

where *τ*_DS_ is the Drude–Smith scattering time and *c* is the backscattering probability ranging from 0 (isotropic scattering) to −1 (complete backscattering).

The intrinsic diffusion length is calculated from *μ* and *t*_2_ as follows:

$$L=\sqrt{\frac{\mu {k}_{{\rm{B}}}T{t}_{2}}{e}},$$

where *k*_B_ is the Boltzmann constant and *T* is the temperature.

We estimate the activation energy (*E*_a_) from the temperature–*γ* relationship using the Arrhenius relation as

$$\gamma =B{{\rm{e}}}^{-({E}_{{\rm{a}}}/{k}_{{\rm{B}}}T\;)},$$

where *B* is the pre-factor.

### TAS

The femtosecond TA setup utilized a regenerative-amplified Ti:sapphire laser system from Coherent and a Helios pump–probe system from Ultrafast Systems. The laser system delivered pulses with a central photon energy of 1.55 eV, a pulse duration of 25 fs and a repetition rate of 1 kHz. The output beam from the amplifier was split into two branches: one beam passed through an optical parametric amplifier (TOPAS-C) to produce pump laser pulses with tunable photon energy, whereas the other beam was focused on a sapphire crystal to create a white-light continuum. The resulting white-light continuum was split into a probe beam and a reference beam. The pump and probe pulses were precisely overlapped both spatially and temporally on the sample. A motorized optical delay line was utilized to adjust the pump–probe delay. The pump pulses were chopped by a mechanical chopper operating at 500 Hz, and the absorbance changes with and without the pump pulse were calculated.

### TAM

The output of a high-repetition-rate amplifier (PH1-20, LIGHT CONVERSION; 800 kHz, 1,030 nm) served as the input to an optical parametric amplifier (TOPAS-Twins, LIGHT CONVERSION) with two independent outputs: one providing the pump beam and the other supplying the probe beam. Both pump and probe beams were spatially filtered. An acousto-optic modulator (Gooch and Housego, AOMO 3080-125) or a mechanical chopper (Stanford Research Systems, SR542) was used to modulate the pump beam at 100 kHz or 1 kHz. A mechanical linear motor stage (Newport, M-IMS600LM-S) was used to control the probe delay with respect to the pump. Both pump and probe beams were focused onto the sample by an objective lens (Nikon, ×10, numerical aperture = 0.25). The transmitted probe beam was collimated through an aspheric lens (numerical aperture = 0.6) and detected by an avalanche photodiode (Thorlabs, APD430A/M). Spatial filters were used to optimize the profile of the beams. The change in probe transmission induced by the pump was detected by a lock-in amplifier (HF2LI, Zurich Instruments). A two-axis galvo mirror (Thorlabs, GVS012/M) was used to scan the probe beam relative to the pump beam in space to image the carrier population in the sample.

## Online content

Any methods, additional references, Nature Portfolio reporting summaries, source data, extended data, supplementary information, acknowledgements, peer review information; details of author contributions and competing interests; and statements of data and code availability are available at 10.1038/s41563-025-02246-2.

## Supplementary information

Supplementary Information Supplementary Figs. 1–18, Supplementary Tables 1–6 and Supplementary Scheme 1.

## Data Availability

All relevant data that support the findings of this study are available within this Article and its Supplementary Information.

## Code availability

All calculations presented in this work are performed using publicly available standard packages. All relevant information used for reproducibility can be found in the Article and its Supplementary Information.

## References

[1] Paul, K. K., Kim, J.-H. & Lee, Y. H. Hot carrier photovoltaics in van der Waals heterostructures. *Nat. Rev. Phys.* **3**, 178–192 (2021).

[2] Liu, C. et al. A hot-emitter transistor based on stimulated emission of heated carriers. *Nature* **632**, 782–787 (2024). 39143208 10.1038/s41586-024-07785-3 PMC11338824

[3] Massicotte, M. et al. Photo-thermionic effect in vertical graphene heterostructures. *Nat. Commun.* **7**, 12174 (2016). 27412308 10.1038/ncomms12174 PMC4947168

[4] Zhou, L. et al. Quantifying hot carrier and thermal contributions in plasmonic photocatalysis. *Science* **362**, 69–72 (2018). 30287657 10.1126/science.aat6967

[5] Yan, J. et al. Dual-gated bilayer graphene hot-electron bolometer. *Nat. Nanotechnol.* **7**, 472–478 (2012). 22659611 10.1038/nnano.2012.88

[6] Tisdale, W. A. et al. Hot-electron transfer from semiconductor nanocrystals. *Science* **328**, 1543–1547 (2010). 20558714 10.1126/science.1185509

[7] Jailaubekov, A. E. et al. Hot charge-transfer excitons set the time limit for charge separation at donor/acceptor interfaces in organic photovoltaics. *Nat. Mater.* **12**, 66–73 (2013). 23223125 10.1038/nmat3500

[8] Yue, S. et al. High ambipolar mobility in cubic boron arsenide revealed by transient reflectivity microscopy. *Science* **377**, 433–436 (2022). 35862517 10.1126/science.abn4727

[9] Guo, Z. et al. Long-range hot-carrier transport in hybrid perovskites visualized by ultrafast microscopy. *Science* **356**, 59–62 (2017). 28386007 10.1126/science.aam7744

[10] Sung, J. et al. Long-range ballistic propagation of carriers in methylammonium lead iodide perovskite thin films. *Nat. Phys.* **16**, 171–176 (2020).

[11] Tielrooij, K.-J. et al. Photoexcitation cascade and multiple hot-carrier generation in graphene. *Nat. Phys.* **9**, 248–252 (2013).

[12] Fu, J. et al. Hot carrier cooling mechanisms in halide perovskites. *Nat. Commun.* **8**, 1300 (2017). 29101381 10.1038/s41467-017-01360-3 PMC5670184

[13] Price, M. B. et al. Hot-carrier cooling and photoinduced refractive index changes in organic–inorganic lead halide perovskites. *Nat. Commun.* **6**, 8420 (2015). 26404048 10.1038/ncomms9420 PMC4598728

[14] Sadasivam, S., Chan, M. K. & Darancet, P. Theory of thermal relaxation of electrons in semiconductors. *Phys. Rev. Lett.* **119**, 136602 (2017). 29341683 10.1103/PhysRevLett.119.136602

[15] Banerji, N., Cowan, S., Vauthey, E. & Heeger, A. J. Ultrafast relaxation of the poly(3-hexylthiophene) emission spectrum. *J. Phys. Chem. C* **115**, 9726–9739 (2011).

[16] Engel, E., Koschorreck, M., Leo, K. & Hoffmann, M. Ultrafast relaxation in quasi-one-dimensional organic molecular crystals. *Phys. Rev. Lett.* **95**, 157403 (2005). 16241760 10.1103/PhysRevLett.95.157403

[17] Illig, S. et al. Reducing dynamic disorder in small-molecule organic semiconductors by suppressing large-amplitude thermal motions. *Nat. Commun.* **7**, 10736 (2016). 26898754 10.1038/ncomms10736 PMC4764867

[18] Noriega, R. et al. A general relationship between disorder, aggregation and charge transport in conjugated polymers. *Nat. Mater.* **12**, 1038–1044 (2013). 23913173 10.1038/nmat3722

[19] Haldar, R. et al. Interplay of structural dynamics and electronic effects in an engineered assembly of pentacene in a metal–organic framework. *Chem. Sci.* **12**, 4477–4483 (2021). 34168750 10.1039/d0sc07073d PMC8179632

[20] Wang, Z., Wang, M., Heine, T. & Feng, X. Electronic and quantum properties of organic two-dimensional crystals. *Nat. Rev. Mater.* **10**, 147–166 (2024).

[21] Skorupskii, G. et al. Efficient and tunable one-dimensional charge transport in layered lanthanide metal–organic frameworks. *Nat. Chem.* **12**, 131–136 (2020). 31767997 10.1038/s41557-019-0372-0 PMC11060427

[22] Dou, J.-H. et al. Atomically precise single-crystal structures of electrically conducting 2D metal–organic frameworks. *Nat. Mater.* **20**, 222–228 (2021). 33230325 10.1038/s41563-020-00847-7

[23] Xie, L. S., Skorupskii, G. & Dinca, M. Electrically conductive metal–organic frameworks. *Chem. Rev.* **120**, 8536–8580 (2020). 32275412 10.1021/acs.chemrev.9b00766 PMC7453401

[24] Wang, M., Dong, R. & Feng, X. Two-dimensional conjugated metal–organic frameworks (2D c-MOFs): chemistry and function for MOFtronics. *Chem. Soc. Rev.* **50**, 2764–2793 (2021). 33465213 10.1039/d0cs01160f

[25] Huang, X. et al. A two-dimensional *π*–*d* conjugated coordination polymer with extremely high electrical conductivity and ambipolar transport behaviour. *Nat. Commun.* **6**, 7408 (2015). 26074272 10.1038/ncomms8408 PMC4490364

[26] Huang, X. et al. Superconductivity in a copper (ii)‐based coordination polymer with perfect kagome structure. *Angew. Chem. Int. Ed.* **57**, 146 (2018). 10.1002/anie.201707568 29160950

[27] Un, H. I. et al. Controlling film formation and host‐guest interactions to enhance the thermoelectric properties of nickel‐nitrogen‐based 2D conjugated coordination polymers. *Adv. Mater.* **36**, 2312325 (2024). 10.1002/adma.202312325 38227294

[28] Sun, L. et al. A microporous and naturally nanostructured thermoelectric metal-organic framework with ultralow thermal conductivity. *Joule* **1**, 168–177 (2017).

[29] Xie, J. et al. Intrinsic glassy-metallic transport in an amorphous coordination polymer. *Nature* **611**, 479–484 (2022). 36289346 10.1038/s41586-022-05261-4

[30] Huang, X. et al. Semiconducting conjugated coordination polymer with high charge mobility enabled by ‘4 + 2’ phenyl ligands. *J. Am. Chem. Soc.* **145**, 2430–2438 (2023). 36661343 10.1021/jacs.2c11511

[31] Dong, R. et al. High-mobility band-like charge transport in a semiconducting two-dimensional metal–organic framework. *Nat. Mater.* **17**, 1027–1032 (2018). 30323335 10.1038/s41563-018-0189-z

[32] Islamov, M. et al. High-throughput screening of hypothetical metal-organic frameworks for thermal conductivity. *npj Comput. Mater.* **9**, 11 (2023).

[33] Lin, W., Canton, S. E., Zheng, K. & Pullerits, T. Carrier cooling in lead halide perovskites: a perspective on hot carrier solar cells. *ACS Energy Lett.* **9**, 298–307 (2024).

[34] Li, N. et al. Cu (i)/Cu (ii) Creutz–Taube mixed‐valence 2D coordination polymers. *Small Methods* **7**, 2201166 (2023). 10.1002/smtd.202201166 36543365

[35] Toyoda, R. et al. Heterometallic benzenehexathiolato coordination nanosheets: periodic structure improves crystallinity and electrical conductivity. *Adv. Mater.* **34**, 2106204 (2022). 10.1002/adma.202106204 35040527

[36] Pan, Z. et al. Synthesis and structure of a non-van-der-Waals two-dimensional coordination polymer with superconductivity. *Nat. Commun.* **15**, 9342 (2024). 39472440 10.1038/s41467-024-53786-1 PMC11522459

[37] Estévez, S. M. et al. Electrical characterization of a large-area single-layer Cu_3_BHT 2D conjugated coordination polymer. *Adv. Funct. Mater.* **35**, 2416717 (2025).

[38] Wang, Z. et al. A Cu_3_BHT‐graphene van der Waals heterostructure with strong interlayer coupling for highly efficient photoinduced charge separation. *Adv. Mater.* **36**, 2311454 (2024). 10.1002/adma.202311454 38381920

[39] Ulbricht, R., Hendry, E., Shan, J., Heinz, T. F. & Bonn, M. Carrier dynamics in semiconductors studied with time-resolved terahertz spectroscopy. *Rev. Mod. Phys.* **83**, 543–586 (2011).

[40] Uhd Jepsen, P. et al. Ultrafast carrier trapping in microcrystalline silicon observed in optical pump–terahertz probe measurements. *Appl. Phys. Lett.* **79**, 1291–1293 (2001).

[41] Hempel, H. et al. Predicting solar cell performance from terahertz and microwave spectroscopy. *Adv. Energy Mater.* **12**, 2102776 (2022).

[42] Zhang, H. et al. Highly mobile hot holes in Cs_2_AgBiBr_6_ double perovskite. *Sci. Adv.* **7**, eabj9066 (2021). 34936431 10.1126/sciadv.abj9066 PMC8694595

[43] Yang, Y. et al. Observation of a hot-phonon bottleneck in lead-iodide perovskites. *Nat. Photon.* **10**, 53–59 (2016).

[44] Zhang, X., Zhou, Y., Cui, B., Zhao, M. & Liu, F. Theoretical discovery of a superconducting two-dimensional metal–organic framework. *Nano Lett.* **17**, 6166–6170 (2017). 28898086 10.1021/acs.nanolett.7b02795

[45] Yang, J. et al. Acoustic-optical phonon up-conversion and hot-phonon bottleneck in lead-halide perovskites. *Nat. Commun.* **8**, 14120 (2017). 28106061 10.1038/ncomms14120 PMC5263885

[46] Smith, N. Classical generalization of the Drude formula for the optical conductivity. *Phys. Rev. B* **64**, 155106 (2001).

[47] Hendry, E., Koeberg, M., Pijpers, J. & Bonn, M. Reduction of carrier mobility in semiconductors caused by charge-charge interactions. *Phys. Rev. B* **75**, 233202 (2007).

[48] Lim, J. W. M. et al. Hot carriers in halide perovskites: how hot truly? *J. Phys. Chem. Lett.* **11**, 2743–2750 (2020). 32183508 10.1021/acs.jpclett.0c00504

[49] Block, A. et al. Tracking ultrafast hot-electron diffusion in space and time by ultrafast thermomodulation microscopy. *Sci. Adv.* **5**, eaav8965 (2019). 31093529 10.1126/sciadv.aav8965 PMC6510559

[50] Gao, G. et al. Unconventional shrinkage of hot electron distribution in metal directly visualized by ultrafast imaging. *Small Methods* **7**, 2201260 (2023). 10.1002/smtd.202201260 36617685

[51] Ginsberg, N. S. & Tisdale, W. A. Spatially resolved photogenerated exciton and charge transport in emerging semiconductors. *Annu. Rev. Phys. Chem.* **71**, 1–30 (2020). 31756129 10.1146/annurev-physchem-052516-050703

[52] Carneiro, L. M. et al. Excitation-wavelength-dependent small polaron trapping of photoexcited carriers in α-Fe_2_O_3_. *Nat. Mater.* **16**, 819–825 (2017). 28692042 10.1038/nmat4936

[53] Liu, J. et al. On-liquid-gallium surface synthesis of ultrasmooth thin films of conductive metal–organic frameworks. *Nat. Synth.* **3**, 715–726 (2024).

[54] Yip, H. K., Schier, A., Riede, J. & Schmidbaur, H. Benzenehexathiol as a template rim for a golden wheel: synthesis and structure of [{CSAu(PPh_3_)}_6_]. *J. Chem. Soc., Dalton Trans.* **15**, 2333–2334 (1994).

