# Supplementary material for: Unveiling high-mobility hot carriers in a two-dimensional conjugated coordination polymer
Source: Nat Mater. 2025 May 13;24(9):1457–64. doi: 10.1038/s41563-025-02246-2 (PMC12404993; doi:10.1038/s41563-025-02246-2)
Supplement: Supplementary file 1 — Supplementary Figs. 1–18, Supplementary Tables 1–6 and Supplementary Scheme 1. [file 41563_2025_2246_MOESM1_ESM.pdf]

nature materials

Supplementary information https://doi.org/10.1038/s41563-025-02246-2

Unveiling high-mobility hot carriers in a two-dimensional conjugated coordination polymer

In the format provided by the authors and unedited

Table of Contents

**Basic characterization**	**2-3**
**Variable-temperature conductivity**	**3**
**Computational details**	**3**
**Supplementary Scheme 1**	**4**
**Supplementary Figures 1-18**	**5-24**
**Supplementary Tables 1-6**	**25-35**
**Supplementary References**	**36-41**

Basic characterization

The structure and properties of the synthesized Cu_3BHT films were studied by various characterization means. Absorption spectra in the ultraviolet-visible and infrared ranges were collected at room temperature using a UV-Vis spectrometer (Cary 5000, Agilent Technologies) and an FT-IR spectrometer equipped with an attenuated total reflectance module (Tensor II, Bruker), respectively. The film thickness was measured by an atomic force microscope (Bruker Multimode 8 HR). The Raman measurements were performed on a Raman microscope (WITec alpha300R) under 532 nm laser excitation. The GIWAXS measurements were performed at beamline P08 at DESY, Hamburg, Germany. The detector used for the measurements was a Perkin Elmer 1620 area detector, placed 707.1 mm behind the sample. The photon beam energy was 18 keV and the beam size was 100 μm (vertical) × 400 μm (horizontal). The sample-to-detector distance and the beam center on the detector were verified using a lanthanum hexaboride (LaB_6) calibration standard. The grazing incidence angle was chosen to be 0.1° and the sample exposure time was 30 s. The scattering data were then analyzed with WxDiff. TEM experiments were performed on an image-side spherical and chromatic aberration-corrected SubÅngström Low-Voltage Electron Microscope (SALVE) instrument operated at 80 kV. The SALVE C_s/C_c corrector adopts a quadrupole-octupole design, which corrects the geometrical axial aberrations up to the 5th-order, off-axial aberrations up to the 3rd-order, and chromatic aberration. Data acquisition was conducted on a Ceta CMOS camera. An objective aperture was inserted to enhance the image contrast in searching mode. The setting of sampling, dose rate, and defocus was conducted in the vicinity of ROI. The acquisition dose for Cu3BHT is 3.2×10^3 eÅ^{-2} (at 80 kV, pixel size: 0.15 Å). The image simulation is conducted by QSTEM (defocus 6 nm). The STEM images were collected using a Thermo Scientific Spectra 300 double-aberration-corrected STEM, operated at 300 kV with a semi-convergence angle of 30 mrad. XPS and UPS measurements were conducted using an AXIS Ultra-DLD ultrahigh vacuum photoemission spectroscopy system (Kratos Co.). For XPS, a monochromatic aluminum K_α source (1,486.6 eV) was employed, while a He I source (21.11 eV) was used for UPS. All measurements were performed under an

ultrahigh vacuum (< 3 × 10 $Torr^{-9}$). During UPS measurements, a negative substrate bias voltage of 9 V was applied. Prior to characterization, the Cu-BHT films were transferred onto highly doped conductive silicon substrates.

Variable-temperature conductivity

The four-terminal transport measurements of longitudinal conductivity were performed using a commercial Physical Property Measurement System. The lock-in method was employed, where an alternating current at a frequency of 17.777 Hz was supplied to the device via a Stanford Research System 830 lock-in amplifier source meter. The voltage was measured by the same instrument.

Computational details

The relaxation of the unit cell parameters, ionic positions, and electronic properties (band structure and density of states) of Cu_3BHT with different Cu valence state configurations were calculated using density functional theory (DFT) with the Vienna *ab-initio* Simulation Package (VASP) version 5.4.1.[1,2] The electronic wave functions were expanded in a plane-wave basis set with a kinetic energy cutoff of 500 eV. Electron-ion interactions were described using the projector augmented wave (PAW) method[3,4] and the range-separated hybrid exchange-correlation functional of HSE06 form.[5] A Monkhorst-Pack Gamma-centered grid[6] with 2×3×4 dimension was used for K-point sampling of the Brillouin zone for the geometry optimization run. For the band-structure calculations, the K-points were generated using the VASPKIT package[7] with the following setup: Gama-centered grid, input Kmesh-Resolution Value for SCF Calculation of 0.04, and input Kmesh-Resolution Value along K-Path for Band Calculation of 0.04. Grimme-D2 correction was applied for the dispersion interlayer interactions.[8] The mixed Cu^{+}/Cu^{2+} states were achieved using the NELECT tag in the INCAR file.

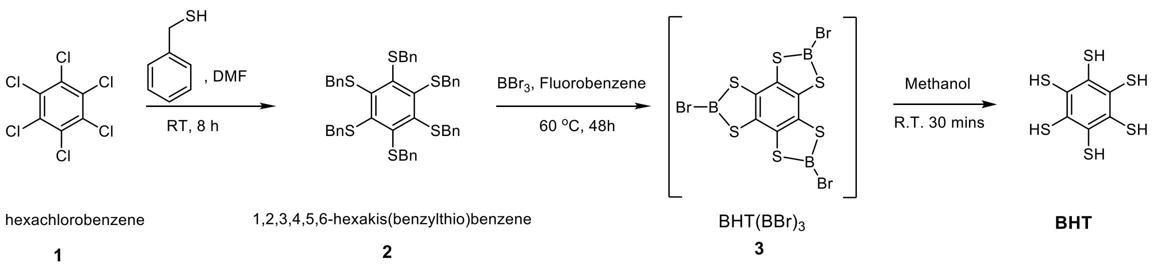

Supplementary Scheme 1. Synthesis of benzenehexathiol (BHT)

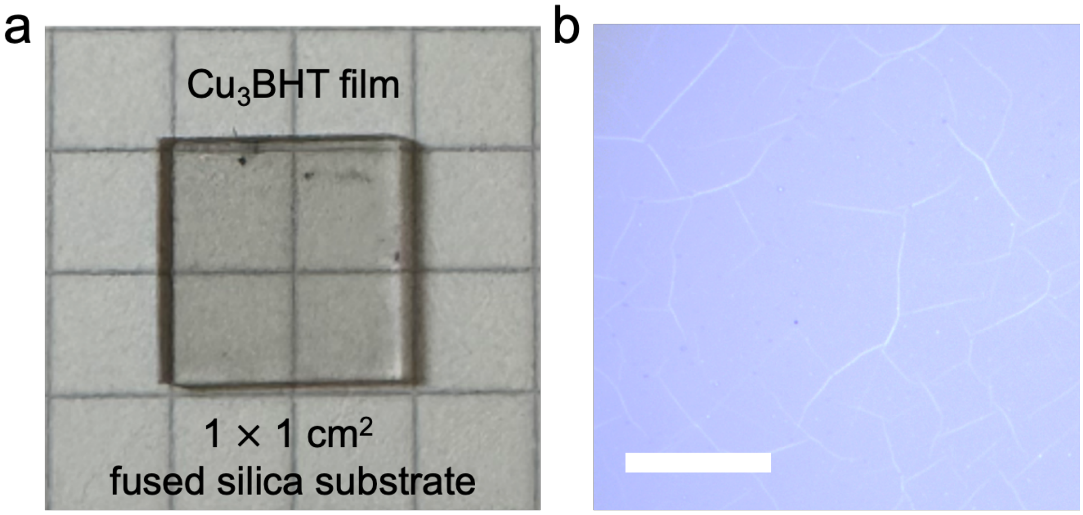

Supplementary Fig. 1 Synthesized Cu_3BHT film (~20 nm thick) transferred on the 1 × 1 cm^2 fused silica substrate. (a) Macroscopic image. (b) Optical image. Scale bar, 20 µm in (b).

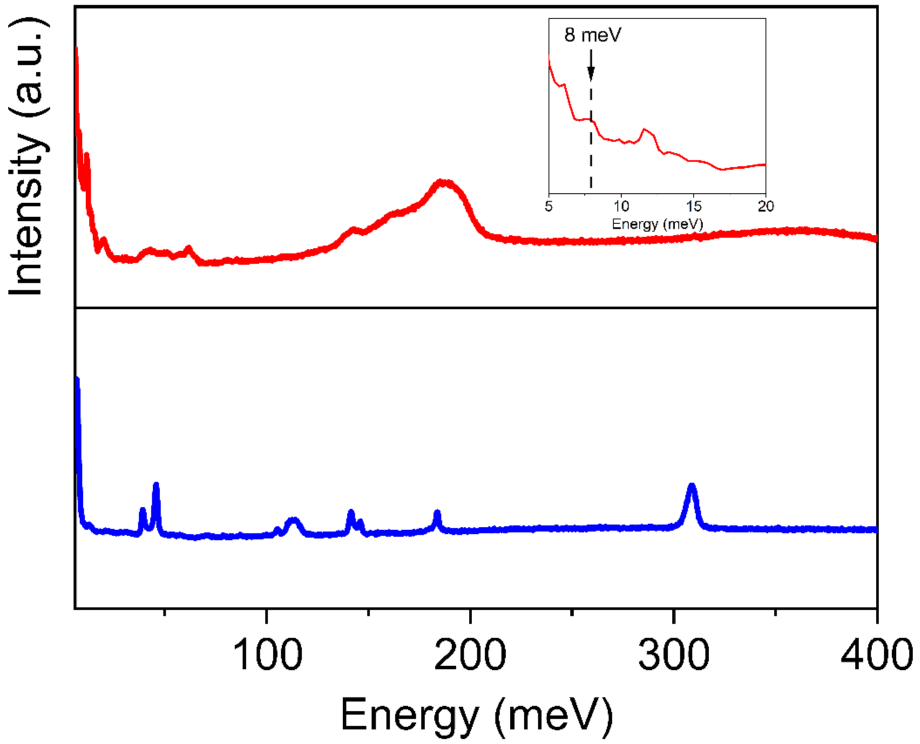

Supplementary Fig. 2 Raman spectra of the synthesized Cu_3BHT film (top) and the BHT ligand (bottom). The inset presents an enlarged view of the 5–20 meV range. The dashed line highlights a Raman peak at 8 meV, which closely aligns with the critical energy inferred from the Arrhenius analysis of the temperature-dependent scattering rate.

The Raman spectrum of the Cu_3BHT film transferred onto a fused silica substrate displays low-energy optical phonon branches around 10 meV and intense bands in the ranges of 30−70 meV and 120−200 meV, attributed to the vibration modes of Cu and S atoms, the interactions between C and S atoms, and the in-plane C stretching, respectively. The absence of the −SH stretching vibration around 310 meV confirms the effective coordination between Cu^{2+} and −SH groups of BHT ligands.

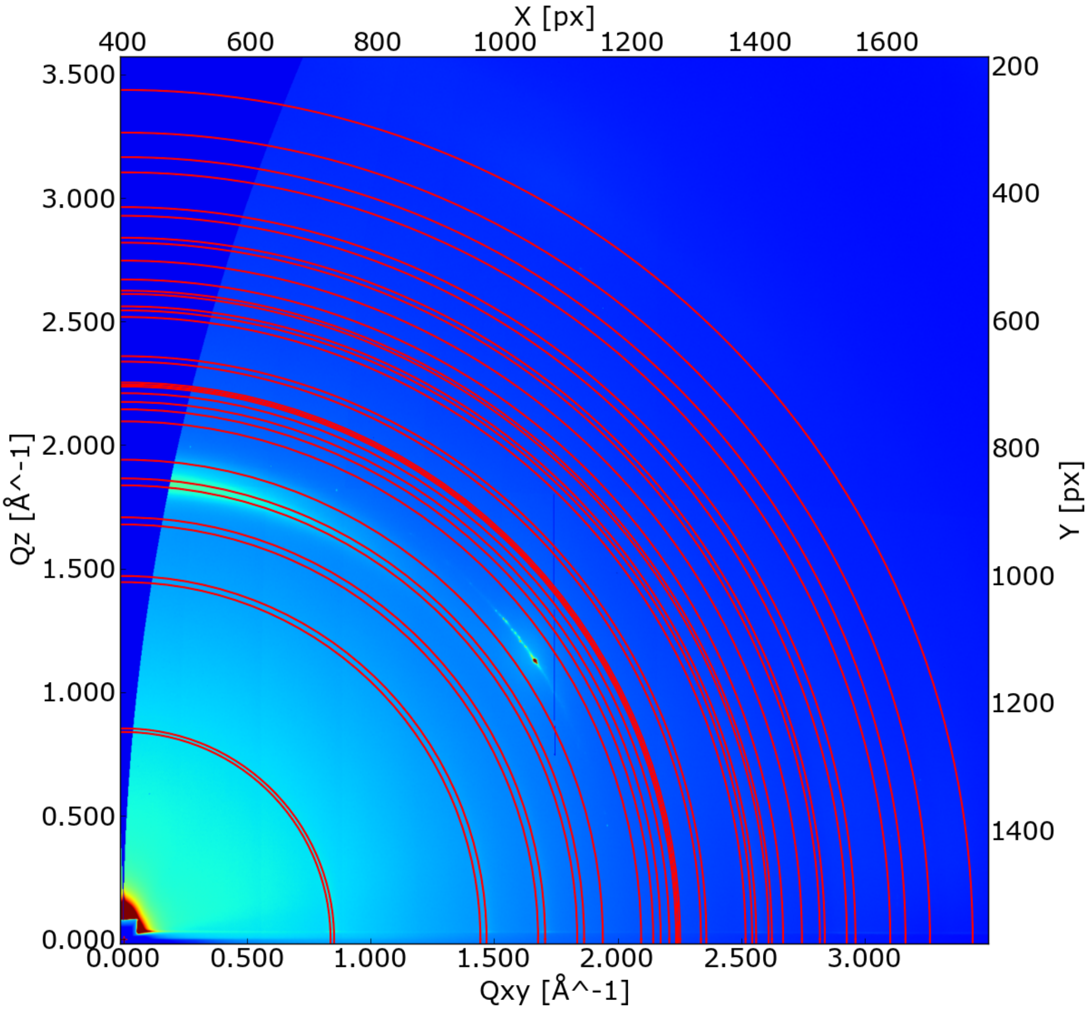

Supplementary Fig. 3 2D GIWAXS image of Cu_3BHT and simulated diffraction signals of the triclinic unit cell, with unit cell parameters of $a = b = 8.675$ Å, $c = 3.489$ Å, $\alpha = \beta = 99.94°$, and $\gamma = 60.12°$.

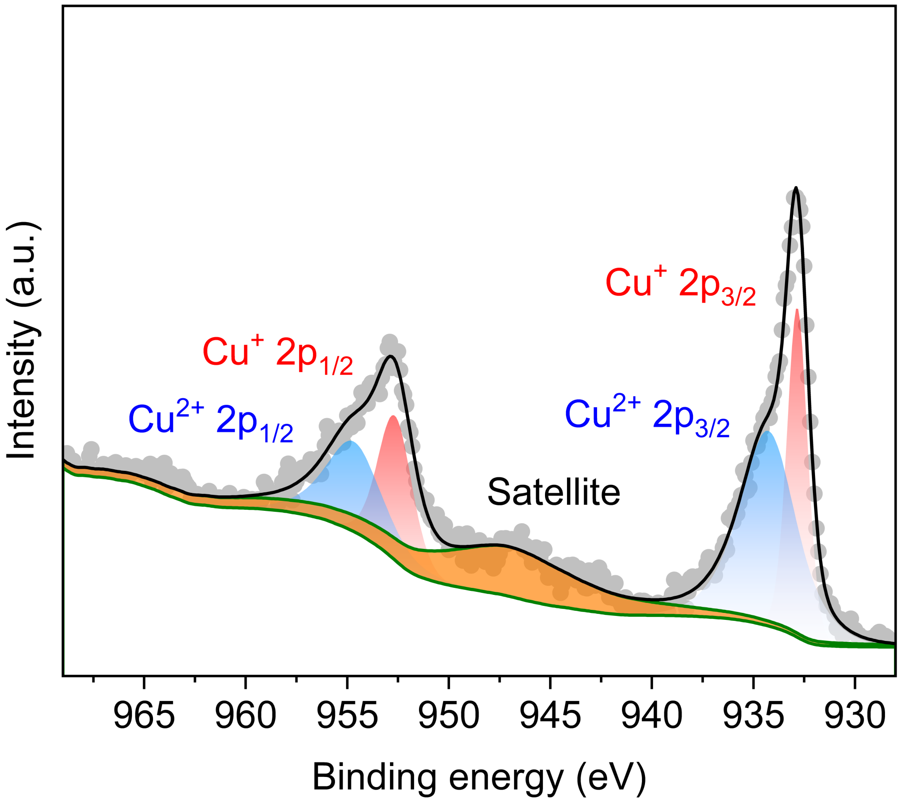

Supplementary Fig. 4 High-resolution X-ray photoelectron spectroscopy (XPS) spectrum of Cu 2*p*.

The high-resolution XPS spectrum of Cu 2*p* shows two asymmetric peaks at 952.9 and 932.9 eV, corresponding to Cu $2p_{1/2}$ and Cu $2p_{3/2}$, respectively, alongside satellite features indicative of Cu^{2+}. Deconvolution of the Cu 2*p* regions reveals the coexistence of Cu^{+} and Cu^{2+} oxidation states.

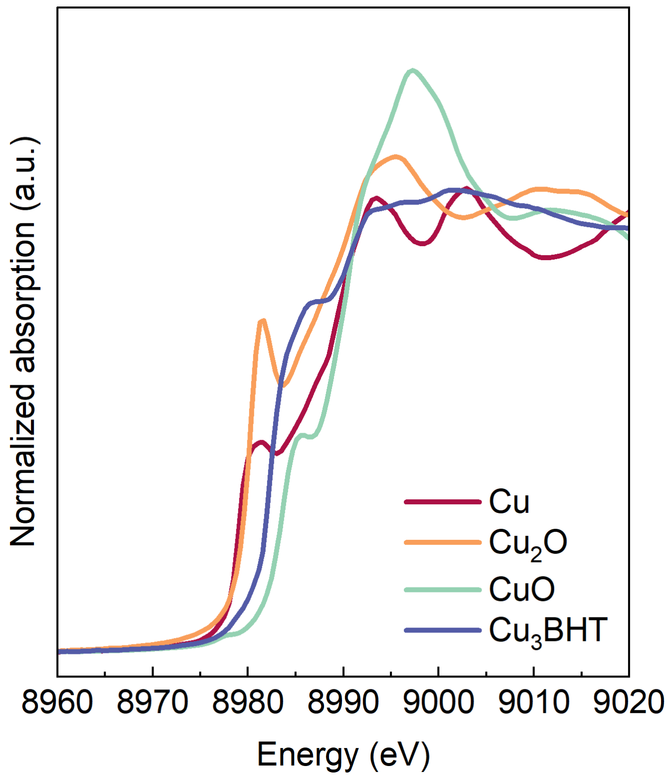

Supplementary Fig. 5 Extended X-ray absorption fine structure (EANES) spectra of Cu, Cu_2O, CuO, and Cu_3BHT.

The XANES spectra corroborate the presence of mixed valence states of Cu, with the K-edge of Cu_3BHT situated between those of Cu_2O and CuO. Notably, the Cu K-edge of Cu_3BHT appears much flatter compared to the well-defined resonances of insulating CuO and Cu_2O, suggesting considerable electron delocalization within the structure.

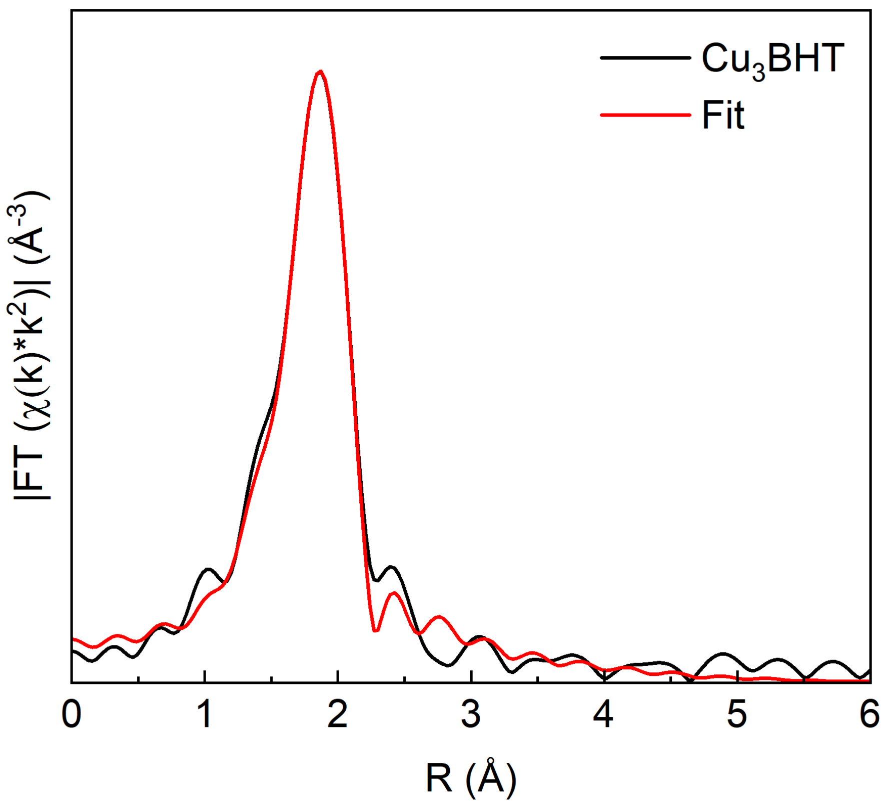

Supplementary Fig. 6 Cu K-edge Fourier-transformed extended X-ray absorption fine structure (EXAFS) *R*-space spectrum of Cu_3BHT. $S0^2$ = 0.876 obtained by fitting the Cu foil is used for the fitting analysis of Cu_3BHT.

The Cu K-edge Fourier-transformed EXAFS *R*-space spectrum showcases a prominent peak at 1.87 Å, attributed to tetra-coordinated Cu-S bonds, along with minor peaks associated with higher shell Cu-C and Cu-Cu scattering. Fitting analysis reveals an average Cu coordination number of 3.22 (± 0.32) and a Cu-S bond length of 2.29 Å, consistent with the square planar coordination geometry.

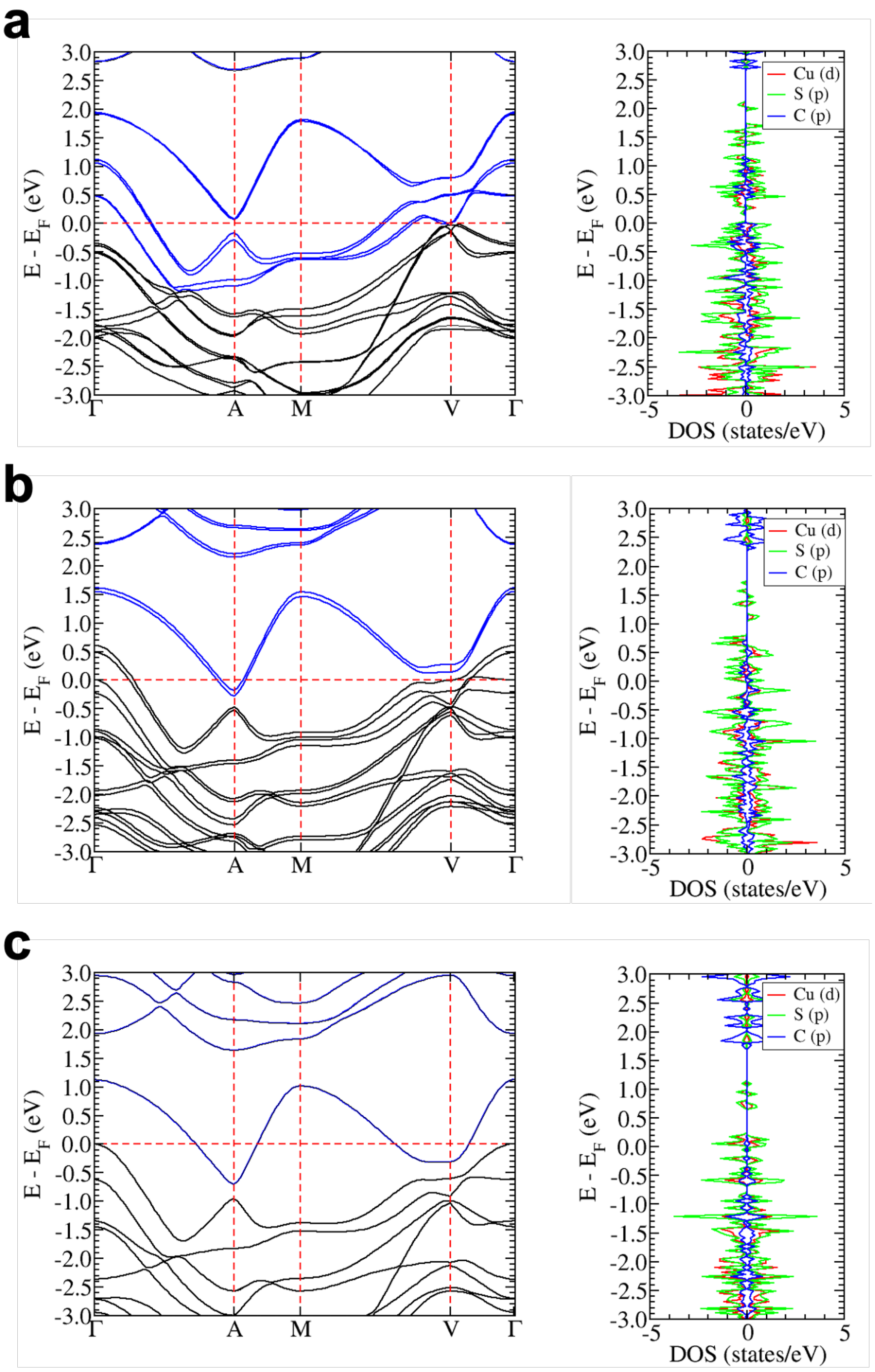

Supplementary Fig. 7 Calculated electronic band structures and projected density of states (DOS) of Cu_3BHT with different Cu valence state configurations. (a) Cu^{2+}/Cu^{+}-ratio = 3:0; (b) Cu^{2+}/Cu^{+}-ratio = 2:1; (c) Cu^{2+}/Cu^{+}-ratio =1:2. Although the Cu^{2+}/Cu^{+}-ratio changes the energy position of the bands, it does not apparently alter the band dispersion. Therefore, the electron and hole masses of Cu_3BHT with different Cu valence configurations can be considered to be similar. Here, taking into account the face-on orientation nature of the as-prepared Cu_3BHT film, we adopt the electron mass of 0.888 m_0 and the hole mass of 0.223 m_0 along the in-plane direction (Cu^{2+}/Cu^{+}-ratio = 0:3, shown in Fig. 1h) to derive the electron–hole reduced effective mass of 0.178 m_0 for mobility estimation.

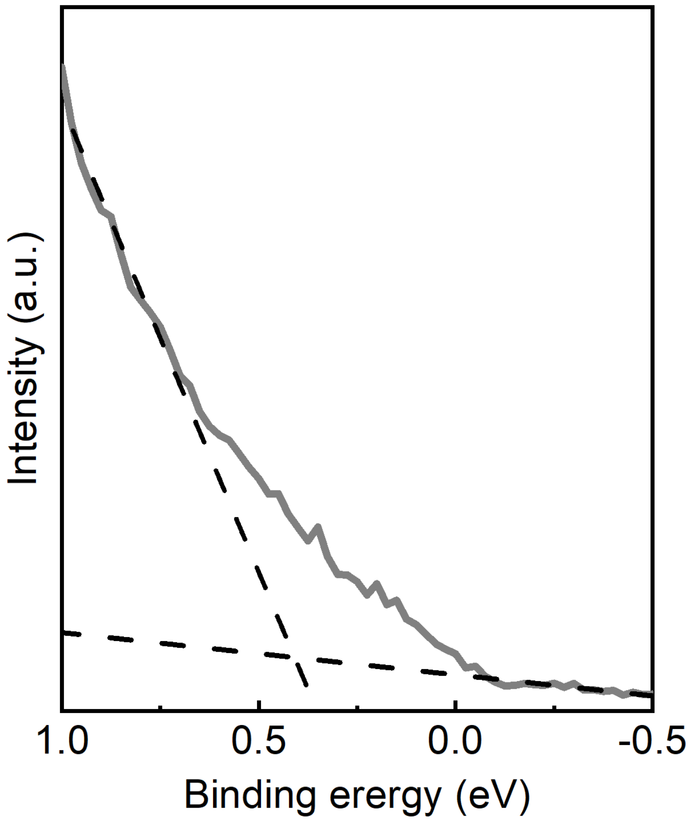

Supplementary Fig. 8 Ultraviolet photoelectron spectroscopy (UPS) of the synthesized Cu_3BHT film.

The UPS spectrum demonstrates an energy separation of ~0.2 eV between the valence band edge and the Fermi level, indicating its semiconducting nature.

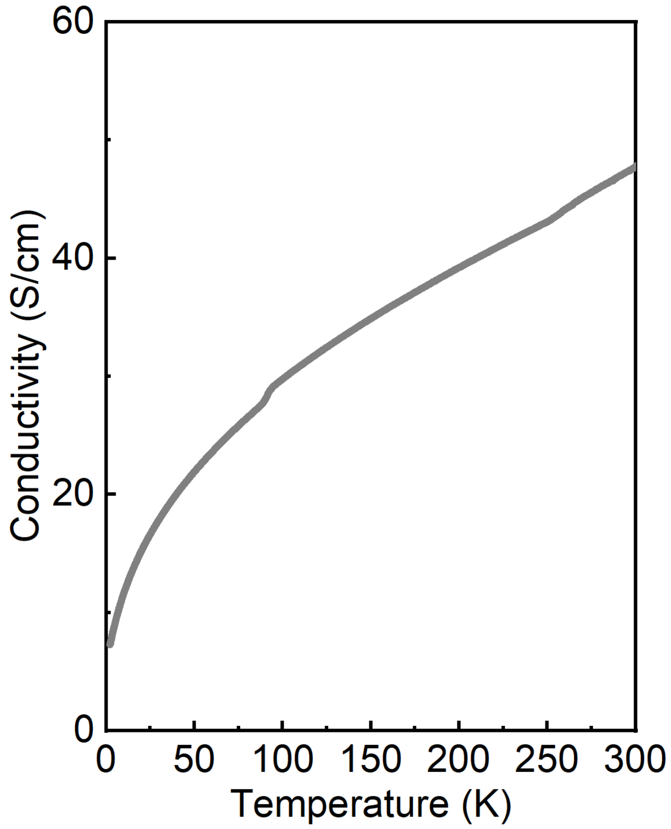

Supplementary Fig. 9 Variable-temperature conductivity measurements of the synthesized Cu_3BHT film from 2 K to 300 K.

Variable-temperature conductivity from 2 K to 300 K using a four-terminal configuration reveals a negative temperature coefficient of conductivity, supporting the semiconducting behavior.

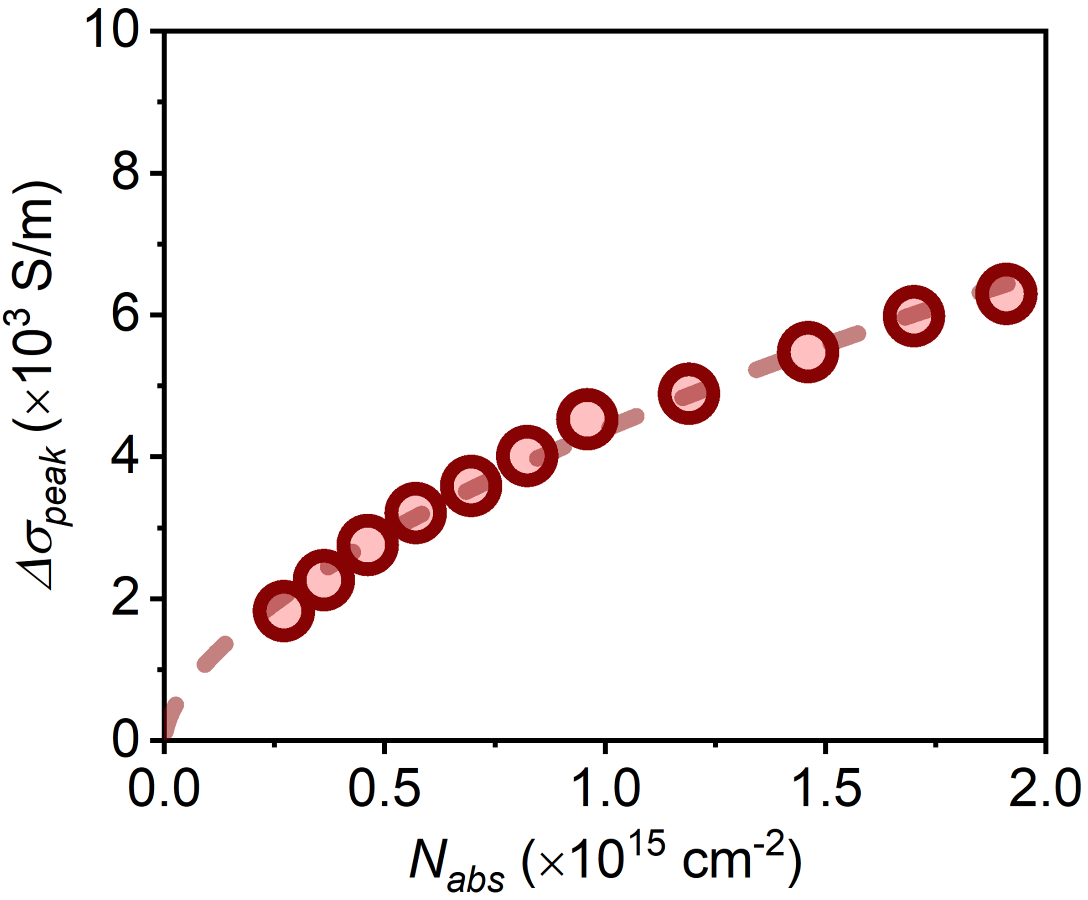

Supplementary Fig. 10 The maximum photoconductivity ($\Delta\sigma_{peak}$) at different absorbed photon densities (N_{abs}) following 1.55 eV excitation.

The maximum photoconductivity $\Delta\sigma_{peak}$ shows a sub-linear dependence on N_{abs}, indicating a decrease in hot carrier mobility at higher N_{abs}. We attribute this observation to increased scattering between hot carriers and hot phonons, driven by the elevated hot phonon population at higher N_{abs}.

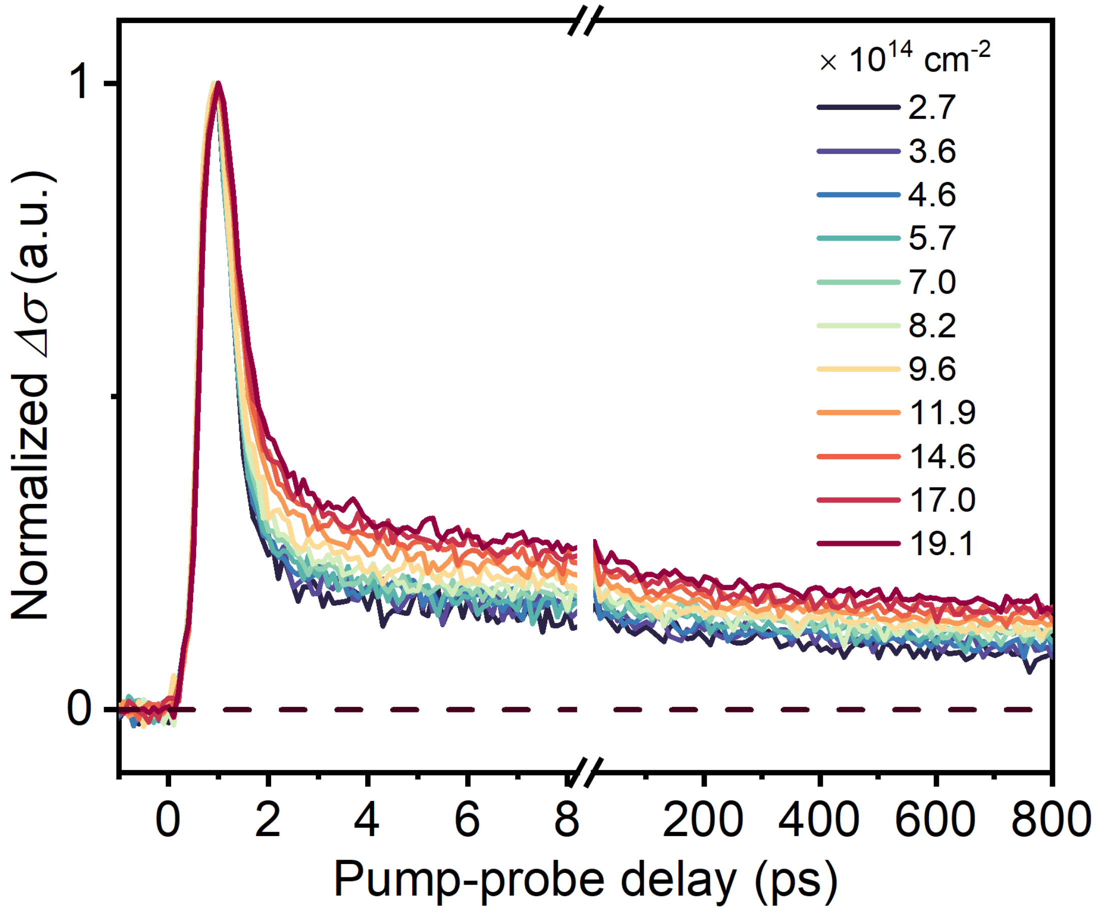

Supplementary Fig. 11 Normalized THz photoconductivity dynamics following 1.55 eV excitation at different N_{abs}.

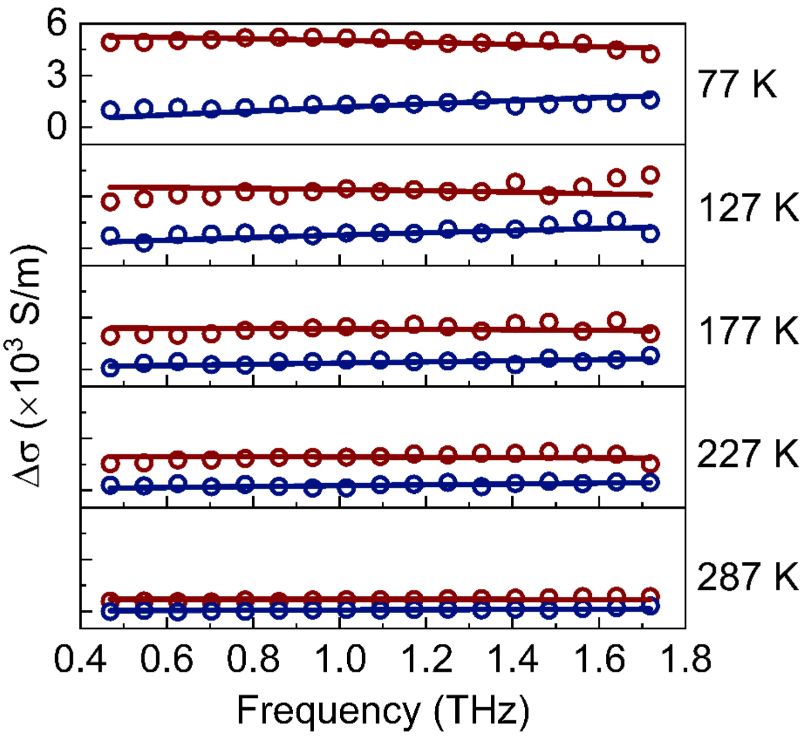

Supplementary Fig. 12 Temperature-dependent complex THz photoconductivity measured in the equilibrium transport regime. The red and blue dots are the real and imaginary components of the complex THz photoconductivity. The red and blue solid lines are the Drude fits to the real and imaginary parts, respectively.

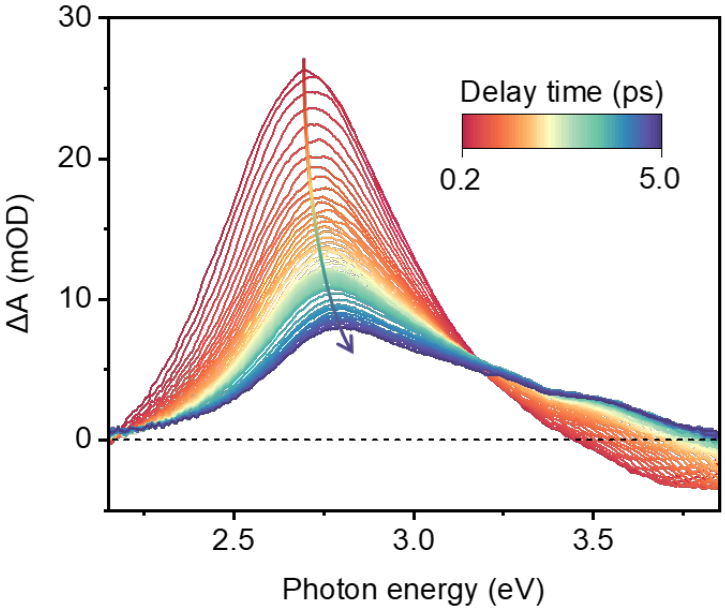

Supplementary Fig. 13 Evolution of TA spectra from 0.2 to 5 ps. The spectral peak exhibits a pronounced blue shift, as indicated by the arrow. This is accompanied by an increase in the high-energy tail. This observation is consistent with the schematic diagram of hot carrier cooling shown in Fig. 4b and the calculated density of states (DOS) in Supplementary Fig. 6.

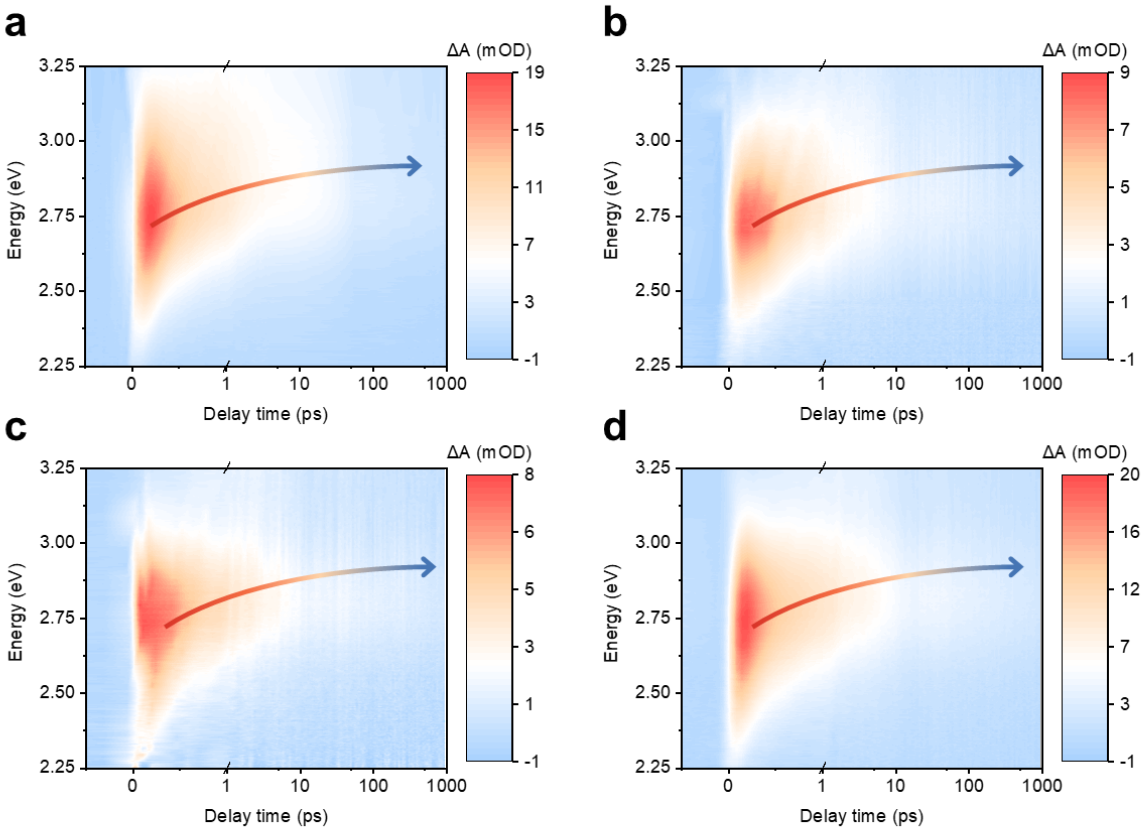

Supplementary Fig. 14 Pseudo-color 2D images under photoexcitation at (a) 3.10 eV, (b) 2.59 eV, (c) 2.11 eV, and (d) 1.77 eV, with N_{abs} = 1.1×10^{15} cm^{-2} for excitations at 3.10 eV and 1.77 eV, and N_{abs} = 0.5×10^{15} cm^{-2} for excitations for 2.59 eV and 2.11 eV. These spectra consistently exhibit a blue shift over time, as indicated by the arrows.

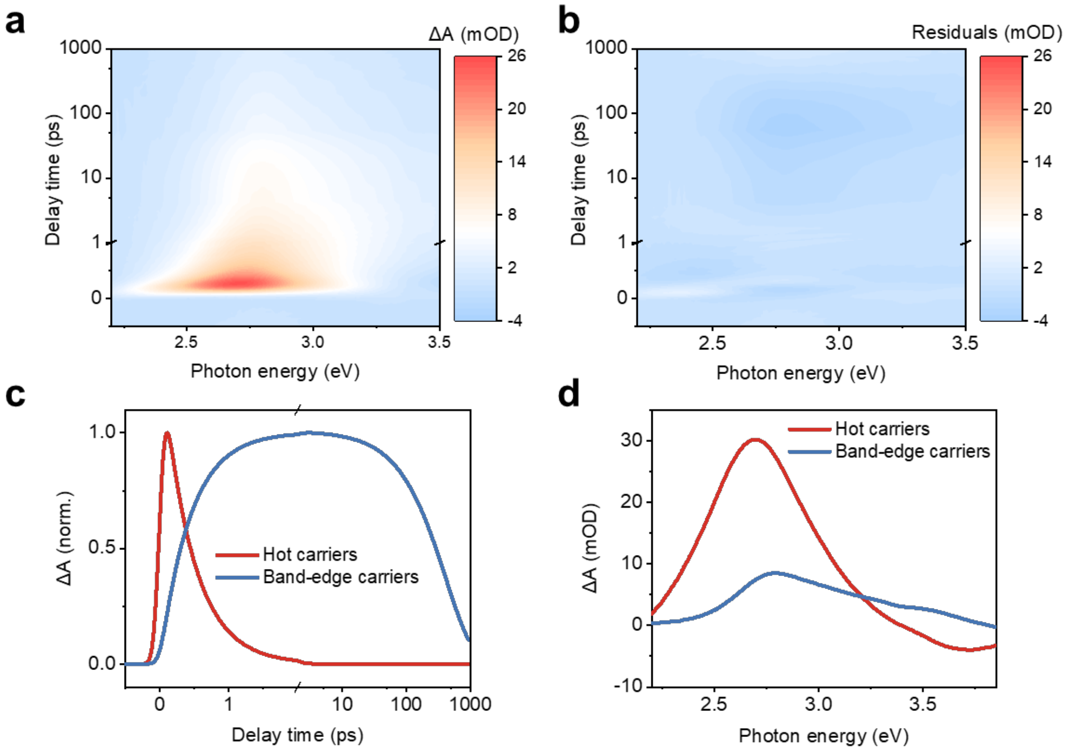

Supplementary Fig. 15 Global fitting analysis of the TA spectra under 1.77 eV photoexcitation. (a) Original TA data. (b) Fitting residuals. (c) The dynamics of hot carriers and band-edge carriers show a cascading trend. (d) Their corresponding spectral signatures.

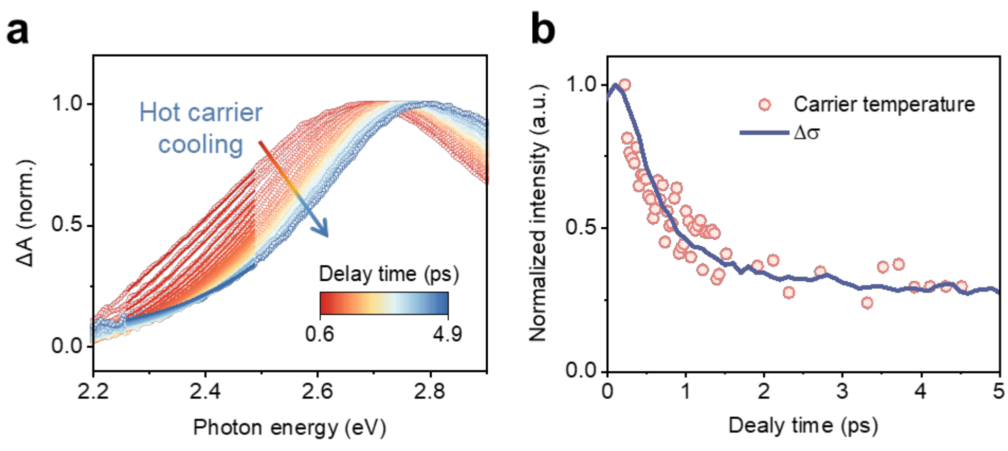

Supplementary Fig. 16 Evaluation of carrier temperature and comparison with THz photoconductivity. (a) Fitting of the low-energy tail following the Fermi-Dirac distribution. Solid lines represent the fitting curves and dots denote the original TA data. (b) Comparison of carrier temperature with THz photoconductivity dynamics.

Broadband TAS has long been a powerful method for evaluating carrier temperatures in semiconducting materials, including III-V semiconductors and perovskites.[9,10] The standard approach typically involves fitting the high-energy tail of the photobleaching (PB) signal with a Fermi-Dirac distribution. In our study, however, we observed only a photoinduced absorption (PIA) signal attributed to hot carrier cooling. Based on the calculated electronic band structure, this PIA signal can be rationalized by transitions from the Cu d-band to the valence band edge in Cu_3BHT. Accordingly, we evaluate the carrier temperature by modeling the low-energy tail of the PIA signal as the high-energy tail of thermalized hot carriers, using the Fermi-Dirac distribution:

$$\Delta A(\hbar\omega) = A_0(\hbar\omega) \cdot \frac{1}{1 + e^{(E_f - \hbar\omega)/k_B T_c}}$$

where E_f is the quasi-Fermi energy level, k_B is the Boltzmann constant, A_0 is a scaling factor, and T_c is the carrier temperature.

We analyze the data starting from 0.2 ps after photoexcitation to ensure that the electronic system has thermalized and can be characterized by a unified T_c (**Supplementary Fig. S16a**). Given that the electronic band structure of Cu_3BHT derivates from a conventional parabolic model, a fully quantitative analysis of carrier temperature may introduce inherent biases. For this reason, we limit our analysis to a

qualitative comparison of the relative trend in carrier temperature with the THz photoconductivity dynamics (**Supplementary Fig. S16b**). The consistent trends indicate that the rapid photoconductivity decay is primarily driven by hot carrier cooling.

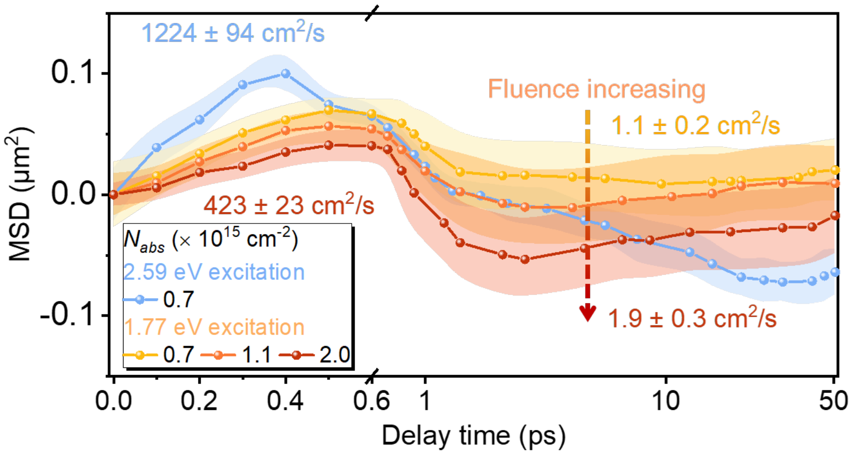

Supplementary Fig. 17 Spatiotemporal profiles of Cu_3BHT under 2.59 and 1.77 eV. Each solid circle represents the variance of the carrier distribution, and the error bars denoted by the shaded areas are the SE obtained from fitting the variance. The shrinkage on the tens-of-picoseconds timescale is attributed to the hot-phonon bottleneck effect. Under 1.77 eV excitation, an increase in the diffusion rate of band-edge carriers is observed with increasing fluence. In contrast, under 2.59 eV excitation, a stronger phonon bottleneck effect suppresses the migration of band-edge carriers.

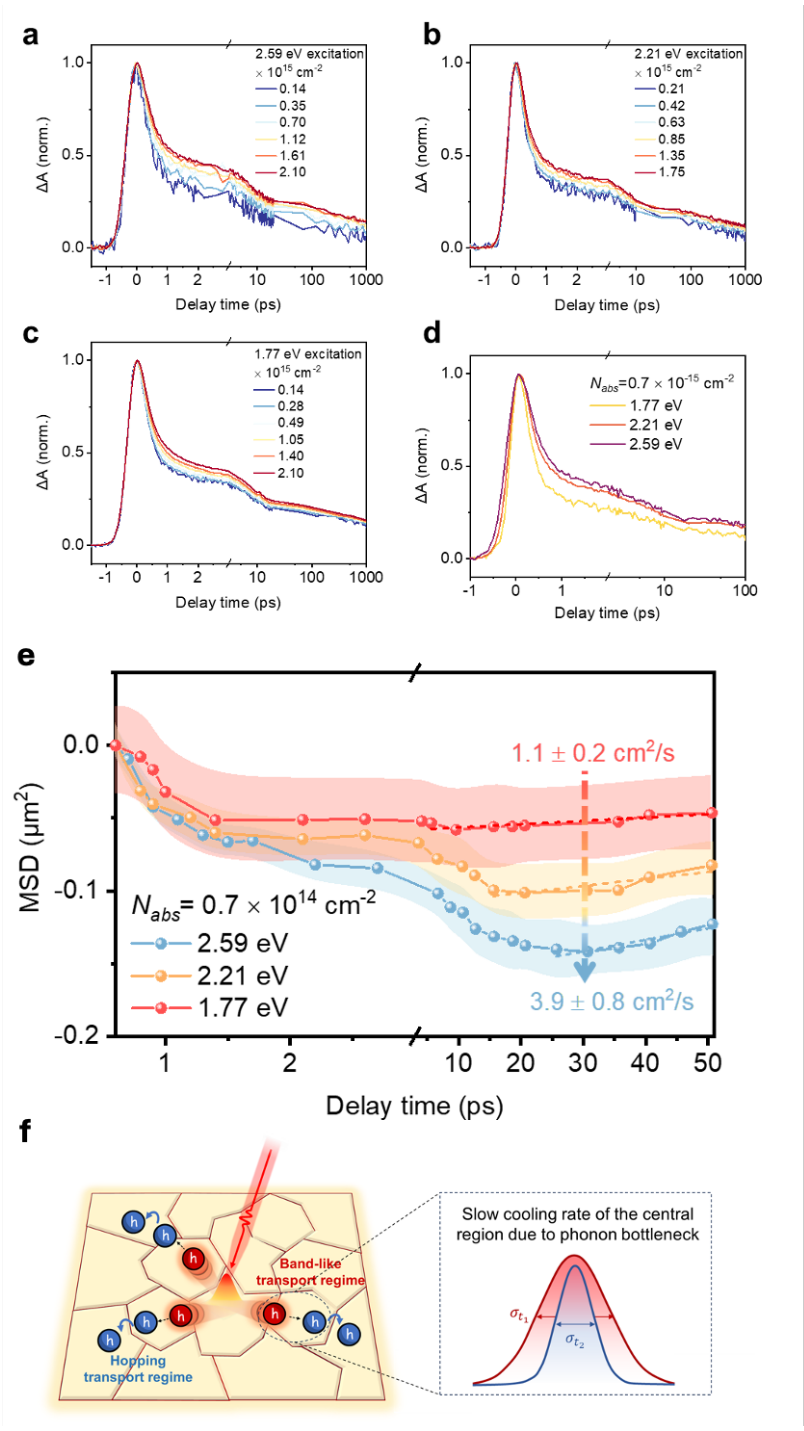

Supplementary Fig. 18 Pump fluence-dependent and pump photon energy-dependent TA dynamics. (a-c) Pump fluence-dependent TA dynamics under photoexcitation at (a) 2.59 eV, (b) 2.21 eV, and (c) 1.77 eV, respectively. (d) Pump-photon energy-dependent TA dynamics at the same N_{abs}. (e) Pump photon energy-dependent spatiotemporal evolution of charge carrier after the fast diffusion phase, at the same N_{abs}. Each solid circle represents the variance of the carrier distribution, and the error bars denoted by the shaded areas are the SE obtained from fitting the variance. The dashed lines are linear fits to the tails of the profiles. (f) Schematic illustration of the spatiotemporal evolution of charge carriers in Cu_3BHT.

Increasing pump fluence (**Supplementary Fig. 18a-c**) or pump photon energy (**Supplementary Fig. 18d**) extends the lifetime of the fast-decay component, signifying a slower hot carrier cooling process due to an intensified hot phonon bottleneck effect. The extended hot carrier cooling lifetime corresponds well to the elongation of the contraction phase in the spatiotemporal evolution of charge carriers (**Supplementary Fig. 18e**). Additionally, at a fixed N_{abs}, increasing the excitation photon energy raises the equilibrated electron–lattice temperature, thereby facilitating thermally activated hopping across grain boundaries. This effect is reflected in the increasing diffusion rates at higher $h\nu$ excitation: 1.1 ± 0.2 cm^2 s^{-1} at 1.77 eV, 2.4 ± 0.8 cm^2 s^{-1} at 2.21 eV, and 3.9 ± 0.8 cm^2 s^{-1} at 2.59 eV. Accordingly, we assign the contraction phase to a consequence of the hot phonon bottleneck, which can be further understood as follows: in the TAM measurements, the Gaussian profile of the pump beam generates a spatially inhomogeneous distribution hot carrier distribution. At the center of the beam, where the hot carrier density is high, hot carrier cooling is suppressed due to the pronounced hot phonon bottleneck effect. Conversely, at the edge of the beam, where hot carrier density is low, hot carrier cooling occurs more rapidly. The spatially asynchronous hot carrier cooling leads to a shrinkage of the spatial distribution of charge carriers, with the magnitude and duration of contraction reflecting the strength of the hot phonon bottleneck effect[11], as schematically illustrated in **Supplementary Fig. 18f**.

Supplementary Table 1. Thermal conductivity and electrical conductivity of selected metals

Materials	Electrical conductivity (S/m)	Thermal conductivity (W/m/k)	Ref.
Aluminum (Al)	3.66×10^{7}	237	[12]
Antimony (Sb)	2.56×10^{6}	24.3	[12]
Barium (Ba)	2.92×10^{6}	18.4	[12]
Beryllium (Be)	2.66×10^{7}	200	[12]
Bismuth (Bi)	9.35×10^{5}	7.87	[12]
Cadmium (Cd)	1.47×10^{7}	96.8	[12]
Calcium (Ca)	2.90×10^{7}	200	[12]
Cesium (Cs)	4.76×10^{6}	35.9	[12]
Chromium (Cr)	7.87×10^{6}	93.7	[12]
Cobalt (Co)	1.79×10^{7}	100	[12]
Copper (Cu)	5.80×10^{7}	401	[12]
Gadolinium (Gd)	7.63×10^{5}	10.5	[12]
Gallium (Ga)	7.35×10^{6}	40.6	[12]
Gold (Au)	4.40×10^{7}	317	[12]
Hafnium (Hf)	2.94×10^{6}	23	[12]
Holmium (Ho)	1.23×10^{6}	16.2	[12]
Indium (In)	1.25×10^{7}	81.6	[12]
Iridium (Ir)	2.13×10^{7}	147	[12]
Iron (Fe)	1.00×10^{7}	80.2	[12]
Lanthanum (La)	1.63×10^{6}	13.4	[12]
Lead (Pb)	4.69×10^{6}	35.3	[12]
Lithium (Li)	1.05×10^{7}	84.7	[12]
Lutetium (Lu)	1.72×10^{6}	16.4	[12]
Magnesium (Mg)	2.22×10^{7}	156	[12]
Manganese (Mn)	6.94×10^{5}	7.82	[12]
Mercury (Hg)	1.04×10^{6}	8.34	[12]
Molybdenum (Mo)	1.81×10^{7}	138	[12]
Nickel (Ni)	1.39×10^{7}	90.7	[12]
Neodymium (Nd)	1.56×10^{6}	16.5	[12]
Niobium (Nb)	6.58×10^{6}	53.7	[12]
Palladium (Pd)	9.26×10^{6}	71.8	[12]
Platinum (Pt)	9.26×10^{6}	71.6	[12]
Polonium (Po)	2.50×10^{6}	20	[12]
Potassium (K)	1.34×10^{7}	102.4	[12]
Rhenium (Re)	5.81×10^{6}	47.9	[12]
Rhodium (Rh)	2.33×10^{7}	150	[12]
Rubidium (Rb)	7.52×10^{6}	58.2	[12]
Silver (Ag)	6.14×10^{7}	429	[12]
Sodium (Na)	2.03×10^{7}	141	[12]
Strontium (Sr)	7.41×10^{6}	35.3	[12]

Tantalum (Ta)	7.41×10^{6}	57.5	[12]
Thallium (Tl)	6.67×10^{6}	46.1	[12]
Tin (Sn)	8.70×10^{6}	66.6	[12]
Tungsten (W)	1.84×10^{7}	174	[12]
Vanadium (V)	4.95×10^{6}	30.7	[12]
Yttrium (Y)	1.68×10^{6}	17.2	[12]
Zinc (Zn)	1.65×10^{7}	116	[12]
Zirconium (Zr)	2.31×10^{6}	22.7	[12]

Supplementary Table 2. Thermal conductivity and electrical conductivity of selected ceramics

Materials	Electrical conductivity (S/m)	Thermal conductivity (W/m/k)	Ref.
Aluminum nitride	~1×10^{-12}	300	[13]
Boron nitrate	~1×10^{-12}	380	[13]
Zinc oxide	~10^{3}	50	[13]
β-SiC	~10^{4}	120	[13]
β-Si_3N_4	1×10^{-11}	200	[13]
SC-20-1	770	37.9	[14]
SC-20-2	480	25.9	[14]
SC-20-3	300	14.1	[14]
SC-20-4	170	5.8	[14]
SC-19-1	500	26	[14]
SC-19-2	380	16.6	[14]
SC-19-3	260	9.4	[14]
SC-19-4	170	5.3	[14]
SC-18-1	83	20.7	[14]
SC-18-2	63	14.8	[14]
SC-18-3	48	9.1	[14]
SC-18-4	31	4.5	[14]
4Y1Ce	0.52	176	[15]
2.5Y2.5Ce	57	172	[15]
1Y4Ce-1	210	166	[15]
1Y4Ce-2	1.2	136	[15]
1Y4Ce-3	460	180	[15]
HP-AlN	7.35×10^{-9}	191.9	[16]
PL-AlN	2.74×10^{-11}	116.8	[16]
3Y-200	~5×10^{-9}	~56	[17]
3Y-300	~3.3×10^{-7}	~45	[17]
3Y-400	~5×10^{-6}	~39	[17]
3YC-200	~1×10^{-10}	~75	[17]
3YC-300	~1×10^{-9}	~59	[17]
3YC-400	~5×10^{-8}	~48	[17]
3YC-500	~2×10^{-6}	~40	[17]
5Y-200	~3.3×10^{-10}	~75	[17]
5Y-300	~2.5×10^{-8}	~59	[17]
5Y-400	~1.25×10^{-6}	~48	[17]
5YC-300	~4×10^{-11}	~59	[17]
5YC-400	~2.5×10^{-9}	~48	[17]
5YC-500	~1×10^{-7}	~40	[17]
5YC-600	~2~$\times10^{-6}$	~35	[17]
10Y-200	~1.11×10^{-12}	~70	[17]
10Y-300	~6.7×10^{-11}	~55	[17]

10Y-400	~3.3×10^{-9}	~45	17
10Y-500	~1.43×10^{-7}	~38	17
10Y-600	~3.3×10^{-6}	~32	17
SC0	5.56×10^{-7}	32	18
SC2	4.55×10^{-5}	115	18
SC4	9.09×10^{-4}	47	18
CVD-SiC	8.33×10^{-8}	132	18
Al_2O_3	~7.8×10^{-6}	~28	19
AS3c	~1×10^{-5}	~30	19
AS5c	~1.5×10^{-5}	~31	19
AS10c	~1×10^{-3}	~34	19
AS15c	~6×10^{-3}	~36	19
AS20c	~4.05×10^{-2}	~30	19
AS3f	~1×10^{-5}	~38	19
AS5f	~1.5×10^{-5}	~31	19
AS10f	~1×10^{-3}	~33	19
AS15f	~1×10^{-2}	~36	19
AS20f	~3×10^{-2}	~39	19

Supplementary Table 3. Thermal conductivity and electrical conductivity of selected polymers

Materials	Electrical conductivity (S/m)	Thermal conductivity (W/m/k)	Ref.
Nylon 12	5.95×10^{-13}	2.96	[20]
Nylon 6	2.33×10^{-14}	3.04	[20]
Nylon 66	8.33×10^{-14}	1.1	[20]
Nylon 46	4.37×10^{-13}	0.301	[20]
Nylon 612	6.76×10^{-13}	0.233	[20]
Nylon 11	4.08×10^{-13}	0.253	[20]
Acetal copolymer	5.65×10^{-14}	0.307	[20]
Polyetheretherketone	9.43×10^{-15}	0.294	[20]
Polybutylene terephthalate	6.33×10^{-15}	0.188	[20]
Polycarbonate	2.04×10^{-15}	0.222	[20]
Polyethylene terephthalate	1.91×10^{-15}	0.258	[20]
Epoxy	1.38×10^{-14}	0.468	[20]
Acetal homopolymer	1.82×10^{-13}	0.311	[20]
PEKK	3.06×10^{-14}	0.25	[20]
Polypropylene copolymer	1.04×10^{-14}	0.398	[20]
Acrylonitrile styrene	4.26×10^{-13}	0.17	[20]
Polystyrene	1.1×10^{-15}	0.17	[20]
Polysulfone	6.58×10^{-15}	0.267	[20]
Polyphenylsulfone	1.69×10^{-14}	0.321	[20]
Polyether block amide	1.26×10^{-11}	0.183	[20]
Poly(P-Xylylene)	9.62×10^{-16}	0.102	[20]
Polyphenylene sulfide	1.16×10^{-14}	0.264	[20]
Polyetherimide	4.1×10^{-15}	0.328	[20]
Polyethersulfone	1.1×10^{-14}	0.473	[20]
Polyvinyl chloride	1×10^{-14}	0.21	[20]
Poly methyl methacrylate	1×10^{-13}	0.21	[20]
Bisphenol-A-diglycidyl-ether	1×10^{-14}	0.19	[13]
Polyvinyl butyral	1×10^{-11}	0.224	[13]
cis-polyacetylene	1×10^{-8}	0.21	[21,22]
trans-polyacetylene	1×10^{-3}	0.38	[21,22]
Polyaniline	1×10^{-5}	0.16	[23]
Poly[Na_x(Ni-ett)]	122	~0.17	[24]
Poly[K_x(Ni-ett)]	112	~0.18	[24]

Poly[Cu_x(Cu-ett)]	76	~0.34	[24]
PDVB	9×10^{-10}	0.579	[25]
PANI-C_{60}-PDVB	63.7	0.164	[25]
Boron nitride-PVB	1×10^{-12}	0.5194	[13]
Zinc oxide- PVB	1×10^{-8}	0.2995	[13]
Silicon carbide-PVB	1×10^{-9}	0.2865	[13]
PMMA/CF	1.07×10^{-2}	0.263	[26]
PMMA/CB	9	0.245	[26]
Epoxy/ZnO/CB	2.5×10^{-6}	0.26	[26]
PS/Fe	1.7×10^{-6}	1.4	[26]
PEDOT:Tos	2.4×10^{4}	0.86	[27]
PH750	5.7×10^{4}	0.34	[28]
PH500	3.3×10^{4}	0.32	[28]
P(MeOPV-*co*-PV)	270	0.4	[29]
P(EtOPV-*co*-PV)	290	0.66	[29]
P(BuOPV-*co*-PV)	100	0.37	[29]

Supplementary Table 4. Thermal conductivity and electrical conductivity of selected conjugated metal-organic frameworks

Materials	Electrical conductivity (S/m)	Thermal conductivity (W/m/k)	Ref.
$Cu_3(BTC)_2$-TCNQ	7	0.27	[30,31]
$Ni_3(HITP)_2$	5880	0.21	[32]
Cu-dsedt	740	0.22	[33]
Cu(I)-dsedt	3520	0.32	[33]
Ni-HATP	20000	0.37	[34]
Cu_3BHT	250000	0.25	[35,36]
$Cu_4C_6S_6$	27000	0.72	[37]
$Cu_{5.5}C_6S_6$	22000	0.59	[37]
Cu(I)-ETT	8860	0.52	[38]

Supplementary Table 5. A comparison of charge mobilities (μ) and diffusion lengths (L).

	Sample name and description	μ ($cm^2\ V^{-1}\ s^{-1}$)	L (nm)	Ref.
Metal-organic frameworks (MOFs)	Cu_3BHT (hot carriers)	up to 2,000 [a]	~300	**This work**
	Cu_3BHT (band-edge carriers)	405 ± 30 [b]	~1100 ± 300	**This work**
	Cu_3BHT	99 (holes) [c] 116 (electrons) [c]	N/A	39
	Cu_4DHTTB	88 ± 15 [b]	N/A	40
	Ag_4TSHQ	352 ± 15 [d]	N/A	41
	Ag_4TTHQ	137 ± 7 [d]	N/A	42
	Ag_4TTBQ	130 ± 9 [d]	N/A	42
	Cu_3(HF-HH-*c*HBC)$_2$	9.3 ± 1.1 [d]	N/A	43
	Cu_3(HCl-HH-*c*HBC)$_2$	3.3 ± 0.4 [d]	N/A	43
	c-HBC-6O-Cu	~48 [d]	N/A	44
	c-HBC-8O-Cu	~38 [d]	N/A	44
	c-HBC-12O-Cu	~64 [d]	N/A	44
	$K_3Fe_2[PcFe\text{-}O_8]$	15 ± 2 [d]	N/A	45
	$K_3Fe_2[PcFe\text{-}O_8]$	0.1 [e]	N/A	45
	$Fe_3(THT)_2(NH_4)_3$	211 ± 7 [b]	N/A	46
	$Fe_3(THT)_2(NH_4)_3$	229 ± 33 [e]	N/A	46
	$Cu_3(HHB)_2$	2.4 ± 0.3 [e]	N/A	47
	$Ni_3(HITP)_2$	48.6 [c]	N/A	48
	$Ni_3(HITP)_2$	45.4 [c]	N/A	49
	$Ni_2[CuPc(NH)_8]$	1.6 ± 0.2 [e]	N/A	50
	$Fe_2(BDP)_3$	0.02 [f]	N/A	51

	$K_{0.8}Fe_2(BDP)_3$	0.29 [f]	N/A	51
	$K_{0.98}Fe_2(BDP)_3$	0.84 [c]	N/A	51
	[In(isophthalate)$_2$]	4.6×10^{-3} [c]	N/A	52
	Zn_2(TTFTB)	0.2 [f]	N/A	53
	Zn(H_2DPPDB)	3–4 × 10^{-3} [c]	N/A	54
	Zn[Pd(DPPDB)]	2×10^{-3} [c]	N/A	54
Covalent organic frameworks (COFs)	CuPc-MIDA-COF	13.3 ± 7.5 [g]	N/A	55
	CuPc-MIDA-COF	8.3 [e]	N/A	55
	HHTP-MIDA-COF	3.4 ± 2.5 [g]	N/A	55
	H_2P-COF	3.5 [f]	N/A	56
	CuP-COF	0.19 [f]	N/A	56
	ZnP-COF	0.048 [f]	N/A	56
	NiPc-COF	1.3 [f]	N/A	57
	NiPc-BTDA COF	0.6 [f]	N/A	58
	COF-366	8.1 [f]	N/A	59
	COF-66	3 [f]	N/A	59
	2D D-A COF	0.05 [f]	N/A	60
	CS-COF	4.2 [f]	N/A	61
	TTF-Ph-COF	0.2 [f]	N/A	62
	TTF-Py-COF	0.08 [f]	N/A	62
	HBC-COF	0.7 [f]	N/A	63
	CuPc-pz COF	0.9 ± 0.2 [e]	N/A	64
	ZnPc-pz COF	4.8 ± 0.7 [e]	N/A	64
	NiPc-CoTAA	0.15 [e]	N/A	65
	BUCT-COF-1	2.75 ± 0.22 [e]	N/A	66
	DBOV-COF	0.6 ± 0.1 [d]	N/A	67
	sp^2c-COF	22.1 ± 2.7 [d]	N/A	68
	sp^2c-COF-6	2.3 ± 0.5 [d]	N/A	68
	sp^2c-COF-8	< 0.1 [d]	N/A	68

	sp^{2}c-COF-9	5.8 ± 0.9 [d]	N/A	68
	c-HBC-COF	44 [d]	N/A	69
	TPB-TFB COF	165 ± 10 [b]	~130	70
	V-2D-COF-W1	1.4 [d]	N/A	71
	V-2D-COF-W3	10.3 [d]	N/A	71
	V-2D-COF-W4	0.6 [d]	N/A	71
	2DPAV-BDT-BT	65 [d]	N/A	72
	2DPAV-BDT-BP	17 [d]	N/A	72
	2DCP-NiPc	971 ± 44 [b]	~237	73
	2DCP-CuPc	460 ± 31 [b]	~157	73
	AntTTH	~0.1 [f]	N/A	74
	1D-Pery-COF	66 ± 14 [g]	N/A	75
	2D M- Pery-COF	49 ± 10 [g]	N/A	75
	2D PL-Pery-COF	21 ± 4 [g]	N/A	75

[a]Mobility obtained by time-resolved THz spectroscopy, based on the Drude response of band-edge carriers and the photoconductivity ratio between hot carriers and band-edge carriers. [b]Mobility obtained by time-resolved THz spectroscopy, based on the Drude model. [c]Mobility obtained by field-effect measurements. [d]Mobility obtained by time-resolved THz spectroscopy, based on the Drude-Smith model, which considers the backscattering effect. [e]Mobility obtained by Hall effect measurements. [f]Mobility obtained by flash-photolysis time-resolved microwave conductivity; [g]Mobility obtained by time-resolved THz spectroscopy without considering the backscattering effect.

Supplementary Table 6. A comparison of diffusion constants (D).

Sample name	Dominant charge species	D ($cm^2\ s^{-1}$)	Ref.
Cu_3BHT (hot carriers)	hot carriers	677 ± 59–1224 ± 94	**This work**
Cu_3BHT	band-edge carriers	1.1 ± 0.2–1.9 ± 0.3	**This work**
$(MAPbBr_3)_{0.17}(FAPbI_3)_{0.83}$	hot carriers	~2	76
$(MAPbBr_3)_{0.17}(FAPbI_3)_{0.83}$	band-edge carriers	0.18	76
$CsPbBr_3$	band-edge carriers	0.12–0.68	77
LiBr-$CsPbBr_3$	band-edge carriers	0.34–0.85	77
$MAPbI_3$	hot carriers	450 ± 10	78
$MAPbI_3$	band-edge carriers	0.7 ± 0.1	78
p-doped silicon	band-edge carriers	35	79
$CsPbBr_3$ single crystal	band-edge carriers	1	79
$MAPbBr_3$ single crystal	band-edge carriers	0.7	79
$MAPbBr_3$ polycrystal	band-edge carriers	0.16	79
TIPS-Pn	triplet exciton	0.003	79
WS_2 few layer	hot carriers	175–980	80
WS_2 few layer	band-edge carriers	10.5	80
TPPS4 aggregates	excitons	3-6	81
tetracene	singlet exciton	0.85–2.8	82
tetracene	triplet excitons	0.06–0.1	82
9-aGNRs	quasi-free carriers	200	83
9-aGNRs	excitons	20 – 60	83
Au film	hot carriers	100.2 ± 6.2	84
Au film	equilibrated carriers	1.17 ± 0.6	84
Au film	hot carriers	95	85
Au film	equilibrated carriers	1.1	85

References

1. Kresse, G. & Furthmüller, J. Efficient iterative schemes for ab initio total-energy calculations using a plane-wave basis set. *Phys. Rev. B* **54**, 11169 (1996).
2. Kresse, G. & Furthmüller, J. Efficiency of *ab-initio* total energy calculations for metals and semiconductors using a plane-wave basis set. *Comput. Mater. Sci.* **6**, 15-50 (1996).
3. Blöchl, P. E. Projector augmented-wave method. *Phys. Rev. B* **50**, 17953 (1994).
4. Kresse, G. & Joubert, D. From ultrasoft pseudopotentials to the projector augmented-wave method. *Phys. Rev. B* **59**, 1758 (1999).
5. Krukau, A. V., Vydrov, O. A., Izmaylov, A. F. & Scuseria, G. E. Influence of the exchange screening parameter on the performance of screened hybrid functionals. *J. Chem. Phys.* **125**, 224106 (2006).
6. Monkhorst, H. J. & Pack, J. D. Special points for Brillouin-zone integrations. *Phys. Rev. B* **13**, 5188 (1976).
7. Wang, V., Xu, N., Liu, J.-C., Tang, G. & Geng, W.-T. VASPKIT: A user-friendly interface facilitating high-throughput computing and analysis using VASP code. *Comput. Phys. Commun.* **267**, 108033 (2021).
8. Grimme, S. Semiempirical GGA-type density functional constructed with a long-range dispersion correction. *J. Comput. Chem.* **27**, 1787-1799 (2006).
9. Li, H. *et al.* Enhanced hot-phonon bottleneck effect on slowing hot carrier cooling in metal halide perovskite quantum dots with alloyed A-site. *Adv. Mater.* **35**, 2301834 (2023).
10. Jang, D.-J. *et al.* Energy relaxation of InN thin films. *Appl. Phys. Lett.* **91**, 092108 (2007).
11. Wei, X. *et al.* Unveiling spatiotemporal diffusion of hot carriers influenced by spatial nonuniform hot phonon bottleneck effect in monolayer MoS_2. *Nano Lett.* **24**, 9269-9275 (2024).
12. Haynes, W. M. *CRC Handbook of Chemistry and Physics*. (CRC press, 2016).
13. Alva, G., Lin, Y. & Fang, G. Thermal and electrical characterization of polymer/ceramic composites with polyvinyl butyral matrix. *Mater. Chem. Phys.* **205**, 401–415 (2018).
14. Kultayeva, S., Ha, J.-H., Malik, R., Kim, Y.-W. & Kim, K. J. Effects of porosity on electrical and thermal conductivities of porous SiC ceramics. *J. Eur. Ceram. Soc.* **40**, 996–1004 (2020).
15. Kusunose, T. & Sekino, T. Improvement in fracture strength in electrically conductive AlN ceramics with high thermal conductivity. *Ceram. Int.* **42**, 13183–13189 (2016).
16. Jiang, H. *et al.* Effect of hot-pressing sintering on thermal and electrical properties of AlN ceramics with impedance spectroscopy and dielectric relaxations analysis. *J. Eur. Ceram. Soc.* **39**, 5174–5180 (2019).
17. Kim, H. *et al.* Effects of carbothermal reduction on the thermal and electrical conductivities of aluminum nitride ceramics. *Ceram. Int.* **36**, 2039–2045 (2010).
18. Zhan, G.-D., Mitomo, M. & Mukherjee, A. K. Effects of heat treatment and sintering additives on thermal conductivity and electrical resistivity in fine-

grained SiC ceramics. *J. Mater. Res.* **17**, 2327–2333 (2002).
19. Parchoviansky, M., Galusek, D., Švančárek, P., Sedláček, J. & Šajgalík, P. Thermal behavior, electrical conductivity and microstructure of hot pressed Al2O3/SiC nanocomposites. *Ceram. Int.* **40**, 14421–14429 (2014).
20. https://www.matweb.com/.
21. Moses, D. & Denenstein, Ajp. Experimental determination of the thermal conductivity of a conducting polymer: Pure and heavily doped polyacetylene. *Phys. Rev. B* **30**, 2090 (1984).
22. Epstein, A. J., Rommelmann, H., Abkowitz, M. & Gibson, H. W. Frequency dependent conductivity of polyacetylene. *Mol. Cryst. Liq.* **77**, 81–96 (1981).
23. Yan, H., Sada, N. & Toshima, N. Thermal transporting properties of electrically conductive polyaniline films as organic thermoelectric materials. *J. Therm. Anal. Calorim.* **69**, 881–887 (2002).
24. Sun, Y. *et al.* Organic thermoelectric materials and devices based on p-and n-type poly (metal 1, 1, 2, 2-ethenetetrathiolate) s. *Adv. Mater.* **24**, 932–937 (2012).
25. Cheng, X. *et al.* The decoupling electrical and thermal conductivity of fullerene/polyaniline hybrids reinforced polymer composites. *Compos. Sci. Technol.* **144**, 160–168 (2017).
26. Juwhari, H. K., Abuobaid, A., Zihlif, A. M. & Elimat, Z. M. Investigation of thermal and electrical properties for conductive polymer composites. *J. Electron. Mater.* **46**, 5705–5714 (2017).
27. Ushirokita, H. & Tada, H. In-plane thermal conductivity measurement of conjugated polymer films by membrane-based AC calorimetry. *Chem. Lett.* **45**, 735–737 (2016).
28. Scholdt, M. *et al.* Organic semiconductors for thermoelectric applications. *J. Electron. Mater.* **39**, 1589–1592 (2010).
29. Hiroshige, Y., Ookawa, M. & Toshima, N. Thermoelectric figure-of-merit of iodine-doped copolymer of phenylenevinylene with dialkoxyphenylenevinylene. *Synth. Met.* **157**, 467–474 (2007).
30. Talin, A. A. *et al.* Tunable electrical conductivity in metal-organic framework thin-film devices. *Science* **343**, 66–69 (2014).
31. Erickson, K. J. *et al.* Thin film thermoelectric metal–organic framework with high Seebeck coefficient and low thermal conductivity. *Adv. Mater.* **27**, 3453–3459 (2015).
32. Sun, L. *et al.* A microporous and naturally nanostructured thermoelectric metal-organic framework with ultralow thermal conductivity. *Joule* **1**, 168–177 (2017).
33. Cui, Y. *et al.* Thermoelectric properties of metal-(Z)-1, 2-dihydroselenoethene-1, 2-dithiol coordination polymers. *Sci. Bull.* **63**, 814–816 (2018).
34. Un, H. *et al.* Controlling film formation and host-guest interactions to enhance the thermoelectric properties of nickel-nitrogen-based two-dimensional conjugated coordination polymers. *Adv. Mater.* **36**, 2312325 (2024).
35. Huang, X. *et al.* Superconductivity in a copper(II)-based coordination polymer with perfect kagome structure. *Angew. Chem. Int. Ed.* **57**, 146 (2018).
36. Tsuchikawa, R. *et al.* Unique thermoelectric properties induced by intrinsic

nanostructuring in a polycrystalline thin-film two-dimensional metal–organic framework, copper benzenehexathiol. *Phys. Status Solidi A* **217**, 2000437 (2020).
37. Huang, X. *et al.* Highly conducting organic–inorganic hybrid copper sulfides $Cu_xC_6S_6$ (x= 4 or 5.5): ligand-based oxidation-induced chemical and electronic structure modulation. *Angew. Chem. Int. Ed.* **59**, 22602 (2020).
38. Sheng, P. *et al.* A novel cuprous ethylenetetrathiolate coordination polymer: Structure characterization, thermoelectric property optimization and a bulk thermogenerator demonstration. *Synth. Met.* **193**, 1–7 (2014).
39. Huang, X. *et al.* A two-dimensional π–d conjugated coordination polymer with extremely high electrical conductivity and ambipolar transport behaviour. *Nat. Commun.* **6**, 7408 (2015).
40. Huang, X. *et al.* Semiconducting conjugated coordination polymer with high charge mobility enabled by "4+ 2" phenyl ligands. *J. Am. Chem. Soc.* **145**, 2430-2438 (2023).
41. Wu, S. *et al.* Selenium-substitution strategy for enhanced mobility, tunable bandgap, and improved electrochemical energy storage in semiconducting conjugated coordination polymers. *Angew. Chem. Int. Ed.* **64**, e202419865 (2025).
42. Huang, X. *et al.* Control of the hydroquinone/benzoquinone redox state in high-mobility semiconducting conjugated coordination polymers. *Angew. Chem. Int. Ed.* **63**, e202320091 (2024).
43. Jastrzembski, K. *et al.* Tunable crystallinity and electron conduction in wavy 2D conjugated metal–organic frameworks via halogen substitution. *Small* **20**, 2306732 (2024).
44. Xing, G. *et al.* Conjugated nonplanar copper-catecholate conductive metal–organic frameworks via contorted hexabenzocoronene ligands for electrical conduction. *J. Am. Chem. Soc.* **145**, 8979–8987 (2023).
45. Yang, C. *et al.* A semiconducting layered metal-organic framework magnet. *Nat. Commun.* **10**, 3260 (2019).
46. Dong, R. *et al.* High-mobility band-like charge transport in a semiconducting two-dimensional metal–organic framework. *Nat. Mater.* **17**, 1027-1032 (2018).
47. Wang, Z. *et al.* Ultrathin two-dimensional conjugated metal–organic framework single-crystalline nanosheets enabled by surfactant-assisted synthesis. *Chem. Sci.* **11**, 7665-7671 (2020).
48. Wu, G., Huang, J., Zang, Y., He, J. & Xu, G. Porous field-effect transistors based on a semiconductive metal–organic framework. *J. Am. Chem. Soc.* **139**, 1360-1363 (2017).
49. Wang, B., Luo, Y., Liu, B. & Duan, G. Field-effect transistor based on an in situ grown metal–organic framework film as a liquid-gated sensing device. *ACS Appl. Mater. Interfaces* **11**, 35935-35940 (2019).
50. Wang, M. *et al.* Phthalocyanine-based 2D conjugated metal-organic framework nanosheets for high-performance micro-supercapacitors. *Adv. Funct. Mater.* **30**, 2002664 (2020).

51. Aubrey, M. L. *et al.* Electron delocalization and charge mobility as a function of reduction in a metal–organic framework. *Nat. Mater.* **17**, 625-632 (2018).
52. Panda, T. & Banerjee, R. High Charge carrier mobility in two dimensional Indium (III) isophthalic acid based frameworks. *Proc. Nat. Acad. Sci. India Sect. A* **84**, 331-336 (2014).
53. Narayan, T. C., Miyakai, T., Seki, S. & Dincă, M. High charge mobility in a tetrathiafulvalene-based microporous metal–organic framework. *J. Am. Chem. Soc.* **134**, 12932-12935 (2012).
54. Liu, J. *et al.* Photoinduced charge-carrier generation in epitaxial MOF thin films: high efficiency as a result of an indirect electronic band gap? *Angew. Chem. Int. Ed.* **54**, 7441-7445 (2015).
55. Enquan, J. *et al*. Exceptional electron conduction in two-dimensional covalent organic frameworks. *Chem* **7**, 3309–3324 (2021).
56. Xiao, F. *et al*. High-rate charge-carrier transport in porphyrin covalent organic frameworks: switching from hole to electron to ambipolar conduction. *Angew. Chem. Int. Ed.* **51**, 2618–2622 (2012).
57. Chumakov, Y. *et al.* First-principles study of thermoelectric properties of covalent organic frameworks. *J. Electron. Mater.* **45**, 3445–3452 (2016).
58. Xuesong, D. *et al*. An n-channel two-dimensional covalent organic framework. *J. Am. Chem. Soc.* **133**, 14510–14513 (2011).
59. Shun, W. *et al*. Covalent organic frameworks with high charge carrier mobility. *Chem. Mater.* **23**, 4094–4097 (2011).
60. Xiao, F. *et al*. An ambipolar conducting covalent organic framework with self-sorted and periodic electron donor-acceptor ordering. *Adv. Mater.* **24**, 3026–3031 (2012).
61. Guo, J. *et al*. Conjugated organic framework with three-dimensionally ordered stable structure and delocalized π clouds. *Nat. Commun.* **4**, 2736 (2013).
62. Shangbin, J. *et al*. Two-dimensional tetrathiafulvalene covalent organic frameworks: towards latticed conductive organic salts. *Chem. Eur. J.* **20**, 14608–14613 (2014).
63. Dalapati, S. *et al.* Rational design of crystalline supermicroporous covalent organic frameworks with triangular topologies. *Nat. Commun.* **6**, 7786 (2015).
64. Wang, M. *et al*. Unveiling electronic properties in metal–phthalocyanine-based pyrazine-linked conjugated two-dimensional covalent organic frameworks. *J. Am. Chem. Soc.* **141**, 16810–16816 (2019).
65. Yan, Y. *et al*. Conductive metallophthalocyanine framework films with high carrier mobility as efficient chemiresistors. *Angew. Chem. Int. Ed.* **60**, 10806–10813 (2021).
66. Shitao, W. *et al*. A fully conjugated 3D covalent organic framework exhibiting band-like transport with ultrahigh electron mobility. *Angew. Chem. Int. Ed.* **60**, 9321–9325 (2021).
67. Enquan, J. *et al*. A nanographene-based two-dimensional covalent organic framework as a stable and efficient photocatalyst. *Angew. Chem. Int. Ed.* **61**, e202114059 (2022).

68. Enquan J. *et al*. Module-patterned polymerization towards crystalline 2D sp^2-carbon covalent organic framework semiconductors. *Angew. Chem. Int. Ed.* **61**, e202115020 (2022).
69. Guolong, X, *et al*. Nonplanar rhombus and kagome 2D covalent organic frameworks from distorted aromatics for electrical conduction. *J. Am. Chem. Soc.* **144**, 5042–5050 (2022).
70. Shuai, F. *et al*. Outstanding charge mobility by band transport in two-dimensional semiconducting covalent organic frameworks. *J. Am. Chem. Soc.* **144**, 7489–7496 (2022).
71. Yannan, L. *et al*. Vinylene-linked 2D conjugated covalent organic frameworks by Wittig reactions. *Angew. Chem. Int. Ed.* **61**, e202209762 (2022).
72. Yamei, L. *et al*. A thiophene backbone enables two-dimensional poly(arylenevinylene)s with high charge carrier mobility. *Angew. Chem. Int. Ed.* **62**, e202305978 (2023).
73. Wang, M. *et al*. Exceptionally high charge mobility in phthalocyanine-based poly(benzimidazobenzophenanthroline)-ladder-type two-dimensional conjugated polymers. *Nat. Mater.* **22**, 880–887 (2023).
74. Samrat, G. *et al*. Low band gap semiconducting covalent organic framework films with enhanced photocatalytic hydrogen evolution. *J. Mater. Chem. A* **12**, 247–255 (2024).
75. Fu, S. *et al*. Dimensional evolution of charge mobility and porosity in covalent organic frameworks. *Nat. Commun.* **16**, 2219 (2025).
76. Wang, T. *et al.* Protecting hot carriers by tuning hybrid perovskite structures with alkali cations. *Sci. Adv.* **6**, eabb1336 (2020).
77. Lv, J. *et al.* Hot carrier trapping and it's influence to the carrier diffusion in CsPbBr3 perovskite film revealed by transient absorption microscopy. *Adv. Sci.* **11**, 2403507 (2024).
78. Guo, Z. *et al.* Long-range hot-carrier transport in hybrid perovskites visualized by ultrafast microscopy. *Science* **356**, 59-62 (2017).
79. Delor, M., Weaver, H. L., Yu, Q. & Ginsberg, N. S. Imaging material functionality through three-dimensional nanoscale tracking of energy flow. *Nat. Mater.* **19**, 56-62 (2020).
80. Liu, Q. *et al.* Visualizing hot-carrier expansion and cascaded transport in WS_2 by ultrafast transient absorption microscopy. *Adv. Sci.* **9**, 2105746 (2022).
81. Wan, Y., Stradomska, A., Knoester, J. & Huang, L. Direct imaging of exciton transport in tubular porphyrin aggregates by ultrafast microscopy. *J. Am. Chem. Soc.* **139**, 7287-7293 (2017).
82. Wan, Y. *et al.* Cooperative singlet and triplet exciton transport in tetracene crystals visualized by ultrafast microscopy. *Nat. Chem.* **7**, 785-792 (2015).
83. Varghese, S. *et al.* Ultrafast charge and exciton diffusion in monolayer films of 9-armchair graphene nanoribbons. *Adv. Mater.* **36**, 2407796 (2024).
84. Gao, G. *et al.* Unconventional shrinkage of hot electron distribution in metal directly visualized by ultrafast imaging. *Small Methods* **7**, 2201260 (2023).
85. Block, A. *et al.* Tracking ultrafast hot-electron diffusion in space and time by

ultrafast thermomodulation microscopy. *Sci. Adv.* **5**, eaav8965 (2019).
